# Single Domain Antibodies as a Powerful Tool for High Quality Surface Plasmon Resonance Studies

**DOI:** 10.1371/journal.pone.0124303

**Published:** 2015-03-30

**Authors:** Eduardo Antonio Della Pia, Karen L. Martinez

**Affiliations:** Department of Chemistry & Nano-Science Center, University of Copenhagen, Copenhagen, Denmark; CNR, ITALY

## Abstract

Single domain antibodies are recombinantly expressed functional antibodies devoid of light chains. These binding elements are derived from heavy chain antibodies found in camelids and offer several distinctive properties for applications in biotechnology such as small size, stability, solubility, and expression in high yields. In this study we demonstrated the potential of using single domain antibodies as capturing molecules in biosensing applications. Single domain antibodies raised against green fluorescent protein were anchored onto biosensor surfaces by using several immobilization strategies based on Ni^2+^:nitrilotriacetic acid-polyhistidine tag, antibody-antigen, biotin-streptavidin interactions and amine-coupling chemistry. The interaction with the specific target of the single domain antibodies was characterized by surface plasmon resonance. The immobilized single domain antibodies show high affinities for their antigens with K_D_ = 3–6 nM and outperform other antibody partners as capturing molecules facilitating also the data analysis. Furthermore they offer high resistance and stability to a wide range of denaturing agents. These unique biophysical properties and the production of novel single domain antibodies against affinity tags make them particularly attractive for use in biosensing and diagnostic assays.

## Introduction

Single domain antibodies, also referred to as nanobodies (Ablynx) or VhH, were discovered in the serum of camelids by Hamers-Casterman and co-workers in 1993 [1]. They represent a unique type of functional antibodies that lack the light chains, while preserving the antigen-binding properties of conventional antibodies. Single domain antibodies display exclusive properties [2,3,4] and have been shown to have great potential in a variety of basic research work (*i*.*e*. localization of proteins in cells, stabilizing agents in protein structural studies) [3], biotechnological [5,6] and medical applications [7], disease diagnostics [2,8,9], and therapeutics [10]. Single domain antibodies recognize their antigens with high specificity and affinities similar to IgG antibodies, but thanks to their small size (molecular weight ~13 kDa) they can bind epitope inaccessible to twelve times larger standard antibodies (molecular weight ~150 kDa) [4,11]. Nanobodies exist as monomers, have high solubility and have a low tendency to aggregate because hydrophobic residues of the VhH surface are substituted by more hydrophilic residues [2,11]. Single domain antibodies are resistant to extreme pH, heat denaturation, proteolysis, solvents and detergents [12,13,14,15]. They can be produced in high expression yields in heterologous systems such as bacteria or yeast and are very easy to purify and handle [11]. All these properties make them very attractive capturing molecules for biosensor and diagnostic applications. However, upon immobilization, single domain antibodies might present different properties from the ones observed in solution [16] and their potential as capturing agents still needs to be characterized in details.

Over the last years, Surface Plasmon Resonance (SPR) biosensors have been widely used to monitor biomolecular interactions due to their high sensitivity, reproducibility and label-free capabilities. Previous reports based on SPR experiments have determined kinetic binding parameters of single domain antibodies for their antigens [17,18,19] and demonstrated the utility of nanobodies for detection of tumor markers such as prostate specific antigen (PSA) at clinical relevant concentrations [20,21].

Here we present a detailed SPR study of the interaction between a Green Fluorescent Protein (GFP) and a single domain antibody derived from a llama single domain antibody (GFP-Nb) [3,22,23,24]. The GFP:GFP-Nb complex was chosen as model system because the nanobody can serve as a universal adapter, allowing the immobilization of any GFP chimeric protein on biosensor surfaces. In fact cloning, expression and purification of GFP-fusion proteins can be easily accomplished by using one of the many commercially available vectors for production of GFP chimeric proteins [25]. Furthermore by using the fluorescence properties of GFP, the GFP:GFP-Nb system can be used to study localization and dynamic interactions of proteins *in vivo* [3,24]. The 13 kDa GFP-Nb folds in a barrel-shaped structure (2.5 nm x 4.5 nm) and it has been shown to enable efficient separation of GFP-tagged proteins from cell extracts [23,26]. The GFP-Nb recognizes specifically only GFP derivatives such as wild-type GFP, eGFP, Yellow Fluorescent Protein (YFP), eYFP; while it does not bind other red fluorescent proteins derived from Anthozoa (*i*.*e*. Discosoma Red Fluorescent Protein (DsRed), mFruit series) [22,23,27]. The molecular structure of the GFP:GFP-Nb complex has been determined by X-ray crystallography and isothermal titration calorimetry (ITC) experiments have illustrated the thermodynamics of the complex formation in solution [27].

In this study, we have demonstrated and compared several proof-of-principle immobilization strategies of nanobodies on SPR biosensor surfaces, based on directional non-covalent Ni^2+^:nitrilotriacetic acid (NTA)-polyhistidine tag and antibody-antigen interactions, non-covalent randomly-oriented interactions between *in vivo* biotinylated nanobodies and streptavidin, and covalent coupling between the amino groups of the protein and the carboxylic groups of the biosensor surface. By using these immobilization methods we examine the performance of several commercial SPR chips, determine the kinetic binding constants of the single domain antibodies for their antigens on the different surfaces and compare them with those of classical monoclonal antibodies. In addition we illustrate the advantages of the nanobodies both as capturing agents and ligands over other antibodies. We also investigate the stability of the nanobodies to several harsh conditions (high temperature, extreme pH values and high ionic strength).

## Materials and Methods

### Reagents

HEPES, NaCl, EDTA, tween 20, NiCl_2_, glycine, biotinamidohexanoic acid N-hydroxysuccinimide ester (Bt-NHS) and all the materials used for protein expression and purification were purchased from Sigma-Aldrich (Denmark). 1-ethyl-3-(3-dimethylaminopropyl)carbodiimide hydrochloride (EDC), N-hydroxysuccinimide (NHS), ethanolamine-HCl, sensor chips CM5, NTA and CAP and monoclonal anti-polyhistidine antibody were from GE Healthcare (Denmark). Single domain antibodies against GFP presenting a six histidine-tag at the C-terminal were obtained from Chromotek GmbH (Germany) as GFP-Trap, biotin-labelled monoclonal anti-GFP antibodies were from Novus Biologicals (Denmark), monoclonal anti-GFP antibodies were from Invitrogen (Denmark).

### Production of his-eGFP and GFP

The gene encoding his-eGFP and GFP cloned respectively in pET and pJF plasmids and transformed in *E*. *Coli* BL21 (DE3) were obtained from Addgene [28,29]. Cells containing the plasmids were separately inoculated in 10 mL of Luria Bertani Broth (LB-broth) medium supplemented with 100 μg/mL ampicillin and grown overnight at 37°C (250 rpm). The overnight cell cultures were then diluted 1:100 in LB-broth medium supplemented with 100 μg/mL ampicillin and grown at 37°C in shaking flasks (250 rpm). Cultures were grown until the OD600 reached 0.6–0.8 and then protein overexpression was induced by addition of 0.5 mM isopropyl-h-D-thiogalactopyranoside (IPTG) at 30°C for 3 hours. Cells were harvested by centrifugation at 3000 x *g* for 15 min at 4°C; the cell pellet was suspended in 10 mL of cold phosphate buffer saline (PBS) buffer pH 7.4, 1 mM phenylmethanesulfonylfluoride (PMSF) and 1 mg/mL lysozyme. After 30 min incubation on ice, the suspension was first sonicated for 2 min and then centrifuged at 14000 x *g* for 45 min at 4°C. The supernatant was collected and stored at -20°C. The his-eGFP was purified by immobilized metal affinity chromatography (IMAC) on a His-Trap column (GE Healthcare), and gel filtration on a Superdex 200 10/300 GL column (GE Healthcare). The protein solution was applied on the His-Trap column loaded with nickel and equilibrated with 20 mM sodium phosphate pH 7.4, 0.5 M NaCl, 20 mM imidazole. The column was washed with 20 mM sodium phosphate pH 7.4, 0.5 M NaCl, 100 mM imidazole and the protein was eluted with 20 mM sodium phosphate pH 7.4, 0.5 M NaCl, 500 mM imidazole. The protein solution was further purified on a gel filtration column equilibrated with 10 mM sodium phosphate pH 7.4, 50 mM NaCl. Purification of GFP was performed by ion exchange chromatography on a Q Sepharose High Performance (GE Healthcare) developed with a linear gradient of 0–500 mM NaCl in 10 mM tris buffer pH 8.0. Fractions containing GFP were then concentrated by ultrafiltration with Amicon Ultra 15 mL centrifugal filter units 10 kDa MWCO (EMD Millipore, Denmark) and purified on a Superdex 200 10/300 GL column. Degree of purity of both his-eGFP and GFP proteins was evaluated to be at least 95% based upon sodium dodecyl sulfate-polyacrylamide gel electrophoresis (SDS-PAGE) analysis. Protein concentration was spectroscopically determined using an extinction coefficient at 488 nm (ε_488_) of 55 mM^-1^cm^-1^ [30]. The protein solutions were either used immediately or stored at -20°C.

### Biotinylation of the GFP-Nb

Bt-NHS was dissolved in deionized water (18.2 MΩ cm at 25°C) and then added to the GFP-Nb at a molar ratio of 2:1. The reaction mixture was incubated at 25°C for 60 min with shaking and then the nanobody was separated from free Bt-NHS by ultrafiltration with Amicon Ultra 0.5 mL centrifugal filter units (3 kDa MWCO). The final concentration of the protein was evaluated by measuring the sample absorbance at 280 nm and using an extinction coefficient of 27 mM^-1^ cm^-1^.

### SPR experiments

SPR assays were performed using a BIAcore X100 instrument (GE Healthcare) at a constant temperature of 25°C. Several SPR commercial chips from Biacore (CM5, NTA, CAP and CM5 chip functionalized with anti-polyhistidine) were used. Each chip consists of two flow cell; one of them (flow cell 1) is used as a control while the ligand is immobilized on the second one (flow cell 2). Here data are presented as the difference between the signal collected in flow cell 2 and the signal in flow cell 1. At least a 1000-fold range of protein concentrations injected in random order was investigated in each experiment, and all the experiments were replicated at least twice. All sensorgrams were processed and analyzed by using the BIAcontrol and BIAevaluation software (GE Healthcare). Semi-logarithmic curves of the binding equilibrium response versus the analyte concentration were evaluated by non-linear regression of the Hill equation to yield equilibrium dissociation constants (K_D_). Kinetic rate constants for the interaction GFP:GFP-Nb and GFP:anti-GFP were determined by fitting the corrected sensorgrams to a 1:1 binding model (Langmuir isotherm) using the non-linear least-squares data analysis integrated in the BIAevaluation software. Errors were evaluated as the standard deviation of the measurements.

#### NTA Chip

Immobilization on the NTA chip was performed by activating the surface of flow cell 2 with a 60 s injection of 0.5 mM NiCl_2_ in 10 mM HEPES pH 7.4, 0.15 M NaCl, 50 μM EDTA, 0.05% tween 20 (NTA running buffer), stabilizing both the cells for 300 s and then applying the his-tagged proteins diluted in running buffer to the flow cells for 150 s. The dissociation of the ligand from the surface was monitored for 400 s and then the surface was regenerated by a 60 s injection of 10 mM HEPES pH 7.4, 0.15 M NaCl, 0.35 M EDTA, 0.05% tween 20 (NTA regeneration buffer). Flow rate during all the experiments was 10 μl/min. The affinity of the GFP for the GFP-Nb was probed by immobilizing ~3 nM of the ligand (GFP-Nb) on flow cell 2 (binding of 120 s and surface stabilization of 300 s) and then applying the analyte (GFP) on both flow cells for 150 s and recording its dissociation for 400 s. The cycle ended with the regeneration of the surface (60 s injection) and a stabilization period of 300 s. Flow rate was 30 μl/min.

#### CM5 Chip

In order to determine the best conditions for antibodies immobilization, a pH scouting analysis was performed using eight different buffer solutions (pH values ranging from 4.0 to 6.0) and 50 μg/mL antibodies concentration. Anti-polyhistidine antibody and anti-GFP antibody diluted in 10 mM sodium acetate pH 4.6 to a concentration of 5 μg/mL and GFP-Nb diluted to a concentration of 50 μg/mL in 10 mM acetate pH 5.4 were covalently immobilized on the surface of flow cell 2 of different CM5 chips using primary amine-coupling methods. The carboxyl groups of the dextran matrix were activated by a 7 min injection of a 1:1 mixture of 0.4 M EDC and 0.1 M NHS, then the proteins were injected on the surface for 7 min and the remaining NHS-ester active groups were deactivated by a 7 min injection of 1 M ethanolamine HCl pH 8.5. Anti-polyhistidine and anti-GFP antibody were immobilized to a level of ~10000 Resonance Unit (R.U.), while GFP-Nb were immobilized to a level of ~2500 R.U.. The binding experiments started with a 60 s stabilization period in 10 mM HEPES pH 7.4, 0.15 M NaCl, 0.05% tween 20 (CM5 running buffer) that was followed by the capture of the ligand with a 120 s injection and its dissociation was followed for 400 s. The analyte was then injected on both flow cells for 150 s and its dissociation was recorded for 400 s. The cycle ended with the regeneration of the surface (60 s injection of 10 mM glycine pH 2.0) and a stabilization period of 120 s. The flow rate during the experiments was 30 μl/min.

#### CAP chip

With regards the experiments where the affinity of the biotin-tagged proteins for the surface was probed, the “Biotin CAPture reagent” (GE Healthcare) was flowed on flow cell 2 with a 300 s injection at a flow rate of 2 μl/min and stabilized for a period of 300 s. The biotinylated protein diluted in 10 mM HEPES pH 7.4, 0.15 M NaCl, 50 μM EDTA, 0.05% tween 20 (CAP running buffer) was then flowed on both flow cells for 150 s and its dissociation was recorded for 400 s (flow rate was 10 μl/min). The cycle ended with the regeneration of the surface (120 s injection of 6 M guanidine-HCl and 0.25 M NaOH, CAP regeneration buffer) and a stabilization period of 120 s. In the experiments where the affinity of the GFP for the biotinylated ligand was probed, the surface of both the flow cells was activated (300 s injection of CAPture reagent, flow rate 2 μl/min), and then the ligand solution in CAP running buffer was applied to flow cell 2 for 120 s and the surface was stabilized for 300 s (the concentration of the ligand was 5 nM for the GFP-Nb and 60 nM for the anti-GFP). Following immobilization of the ligand, the analyte was injected on both flow cells for 150 s and its dissociation was recorded for 400 s (flow rate was 30 μl/min). The cycle ended with the regeneration of the surface (120 s injection of CAP regeneration buffer) and a stabilization period of 300 s.

#### GFP-Nb stability experiments

In the stability experiments the GFP-Nb were either diluted in NTA running buffer, kept at different temperatures for 30 min and allowed to recover at room temperature for 30 min (temperature range from 25°C to 100°C), or diluted in NTA running buffer corrected to different pH values by adding 37% HCl or 2 M NaOH solution (pH range from 4.0 to 10.5). The GFP was diluted in NTA running buffer containing different concentrations of NaCl (0 mM, 150 mM, 300 mM, 500 mM and 1 M). The GFP-Nb was then immobilized on the activated flow cell 2 of the NTA chip and the same conditions described in the “NTA Chip” sub-section were used to probe the binding of the GFP-Nb to the Ni:NTA surface and the interaction between GFP-Nb and GFP. The experiments were performed using the NTA chip because the biosensor surface could be easily regenerated by complete removal of the nanobodies allowing the re-use of the sensor chip.

## Results and Discussion

### Interaction of GFP-Nb with SPR biosensor surfaces

**Fig 1:**
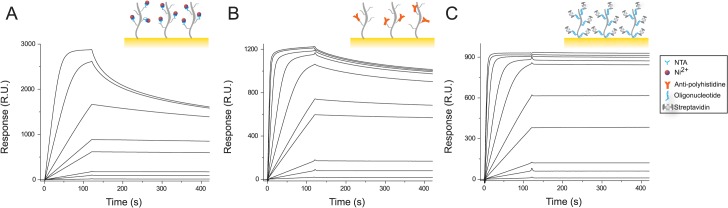
Binding of GFP-Nb to SPR biosensor surfaces. The SPR sensorgrams show the interaction between the GFP-Nb and a NTA surface activated with Ni^2+^ ions (A), a CM5 chip functionalized with anti-polyhistidine antibodies (B) and a CAP-chip sensor surface bearing streptavidin free binding sites (C). Depicted sensorgrams were obtained for GFP-Nb concentration of 1, 5, 10, 35, 50, 100, 250, 500, 1000, 1500 nM (from bottom to top curve).

GFP-Nb was immobilized on three different commercial SPR biosensor chips (Biacore) namely CM5, NTA and CAP chip. The CM5 chip consists of a gold surface covered with a carboxymethyl-terminated dextran layer. The NTA chip is a CM5 chip with pre-immobilized NTA groups where his-tagged molecules can be immobilized *via* Ni^2+^-NTA chelation. The CAP chip consists of a CM5 sensor surface functionalized with single strand DNA (ss-DNA) oligonucleotides to which streptavidin conjugated with the complementary ss-DNA oligonucleotide can be coupled. Biotinylated molecules can then be attached to the biosensor surface. SPR based analysis of the binding of the GFP-Nb to the three different biosensors indicated a strong and specific interaction of the protein for all the surfaces (Fig 1). The SPR traces showed responses ranging from baseline signals (at ~1 nM) to saturation of the surface (at 1 μM). The GFP-Nb showed a very low non-specific binding to the reference surfaces; even at high concentrations the signal measured on the reference surfaces was less than 1% of the signal measured on the respective active surfaces. The response of the chips to the addition of nanobody was very reproducible over different cycles (S1 Fig) and the nanobody could be completely removed from the surface by using the regeneration conditions described in the methods section. However, while the NTA chip allowed running more than a thousand experimental cycles, the CAP and the CM5 chip functionalized with the anti-polyhistidine allowed ~100 cycles due to the degradation of the oligonucleotides and the antibodies immobilized respectively on the two surfaces. Furthermore, due to the presence of a higher number of binding sites, the amount of proteins that could be immobilized on the NTA chip was almost three times the one that could be immobilized on the other two surfaces (Fig 1).

A qualitative analysis of the sensorgrams indicated that the affinity of the GFP-Nb for the surfaces follows the order CAP-chip > anti-polyhistidine > Ni:NTA. In particular, while the dissociation of the biotinylated nanobody from the streptavidin-conjugate surface does not depend on the amount of immobilized protein, the nanobody tends to dissociate from the other surfaces at concentrations higher than 50 nM. Fitting of the SPR sensorgrams to a 1:1 binding model revealed a *k*_on_ = (1.6 ± 0.4) x 10^6^ 1/Ms and a *k*_off_ = (1.2 ± 0.3) x 10^–2^ 1/s for the interaction of the nanobody with the Ni:NTA surface and a *k*_on_ = (1.0 ± 0.3) x 10^6^ 1/Ms and a *k*_off_ = (8.8 ± 1.1) x 10^–4^ 1/s for the interaction of the nanobody with the anti-polyhistidine surface (S2 Fig).

**Fig 2:**
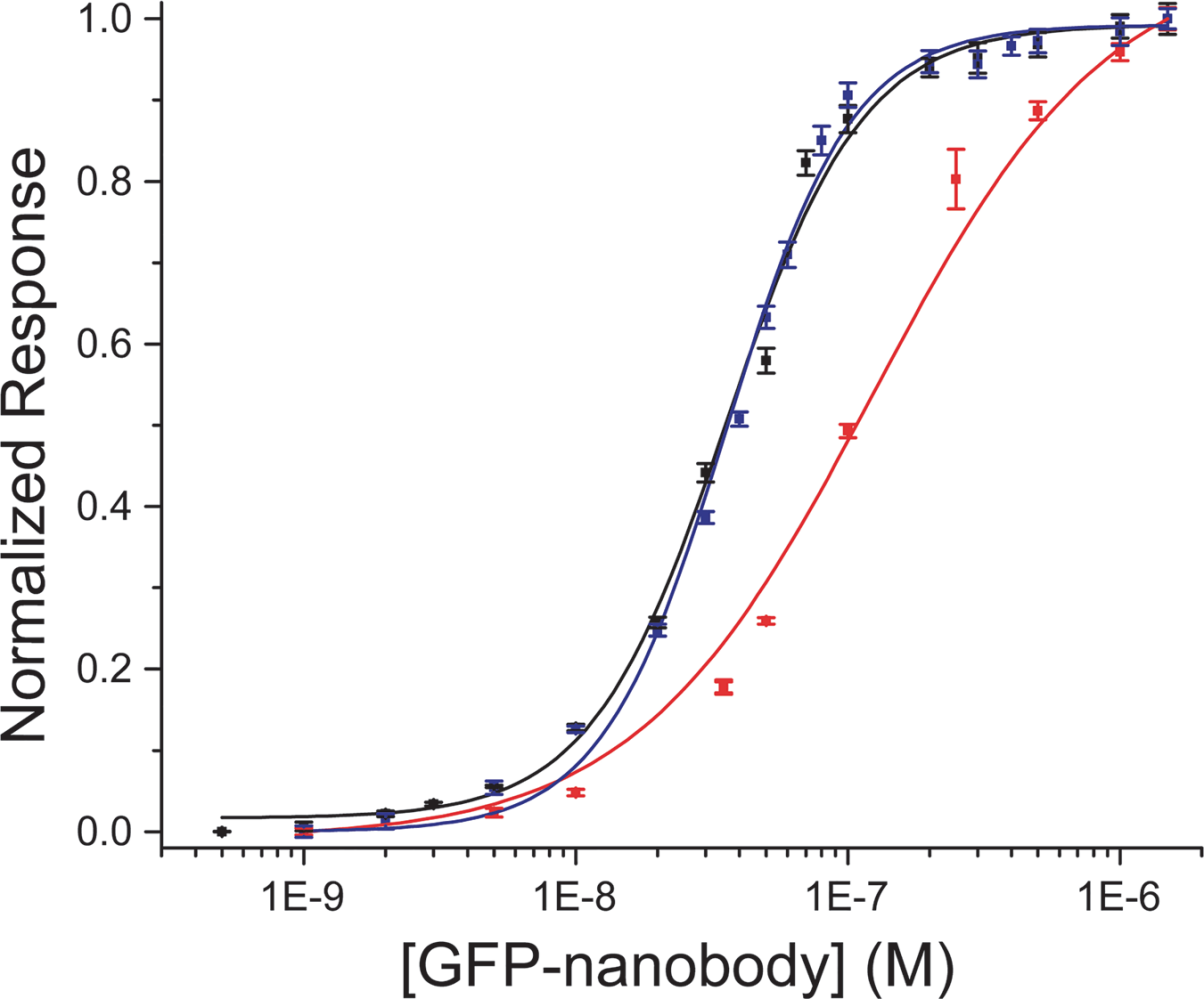
Endpoint response of the reference subtracted binding curves describing the interaction between the GFP-Nb and the different surfaces as function of GFP-Nb concentration. The red, blue and black squares are the raw data for the Ni:NTA, the anti-polyhistidine and the CAP chip respectively, and the lines are the best fit of the data set to the Hill’s equation (R^2^ = 0.99). As choosing a Hill’s coefficient equal to 1 did not produce a good fit of the data describing the interaction between the GFP-Nb and the anti-polyhistidine and the CAP-chip (S2 Fig), a Hill’s coefficient of 2 was set for these lines.

The GFP-Nb end-point response data fitted a simple monovalent interaction with the three surfaces (Fig 2 and S3 Fig). The calculated binding constants were K_D_ = 120 ± 14, 38 ± 3 and 30 ± 5 nM for the Ni:NTA, anti-polyhistidine and streptavidin surface. These values are in close agreement to others previously reported for the interaction of biotin-tagged molecules with streptavidin-functionalized surfaces [31]. However, the GFP-Nb interacts more strongly with the NTA and CM5 SPR chips than another reference polyhistidine-tagged protein (his-eGFP, S1 and S4 Figs). The evaluated equilibrium dissociation constants of his-eGFP were 420 ± 50 nM and 400 ± 33 nM for the Ni:NTA and the anti-polyhistidine surface, respectively. Both the K_D_s are higher than the ones evaluated for the GFP-Nb and this is mainly due to a higher *k*_off_; in particular the *k*_off_ for the anti-polyhistidine surface is (4.8 ± 0.1) x 10^–2^ 1/s (S6 Fig). The differences in affinity could be explained by considering the position of the polyhistidine tag on the C-terminal of the nanobody that is more accessible to the binding sites on the surface than the one of the his-eGFP (S7 Fig).

### The GFP:GFP-Nb complex

**Fig 3:**
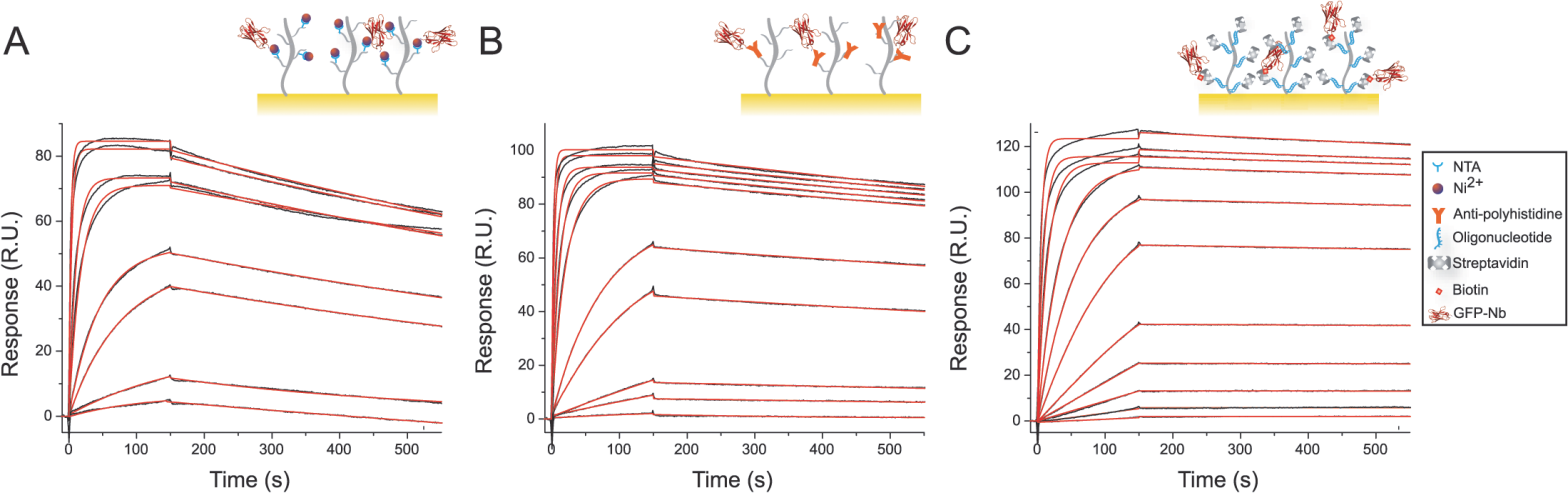
GFP:GFP-Nb complex. Binding of GFP to GFP-Nb immobilized on Ni:NTA (A), anti-polyhistidine antibody on CM5 (B) and streptavidin on CAP chip (C). Black lines are raw data and the red lines are the fitting to a 1:1 binding model. Depicted sensorgrams were obtained for GFP concentration of 0.1, 0.5, 1, 5, 10, 50, 100, 200, 500, 1000 nM (from bottom to top curve).

**Fig 4:**
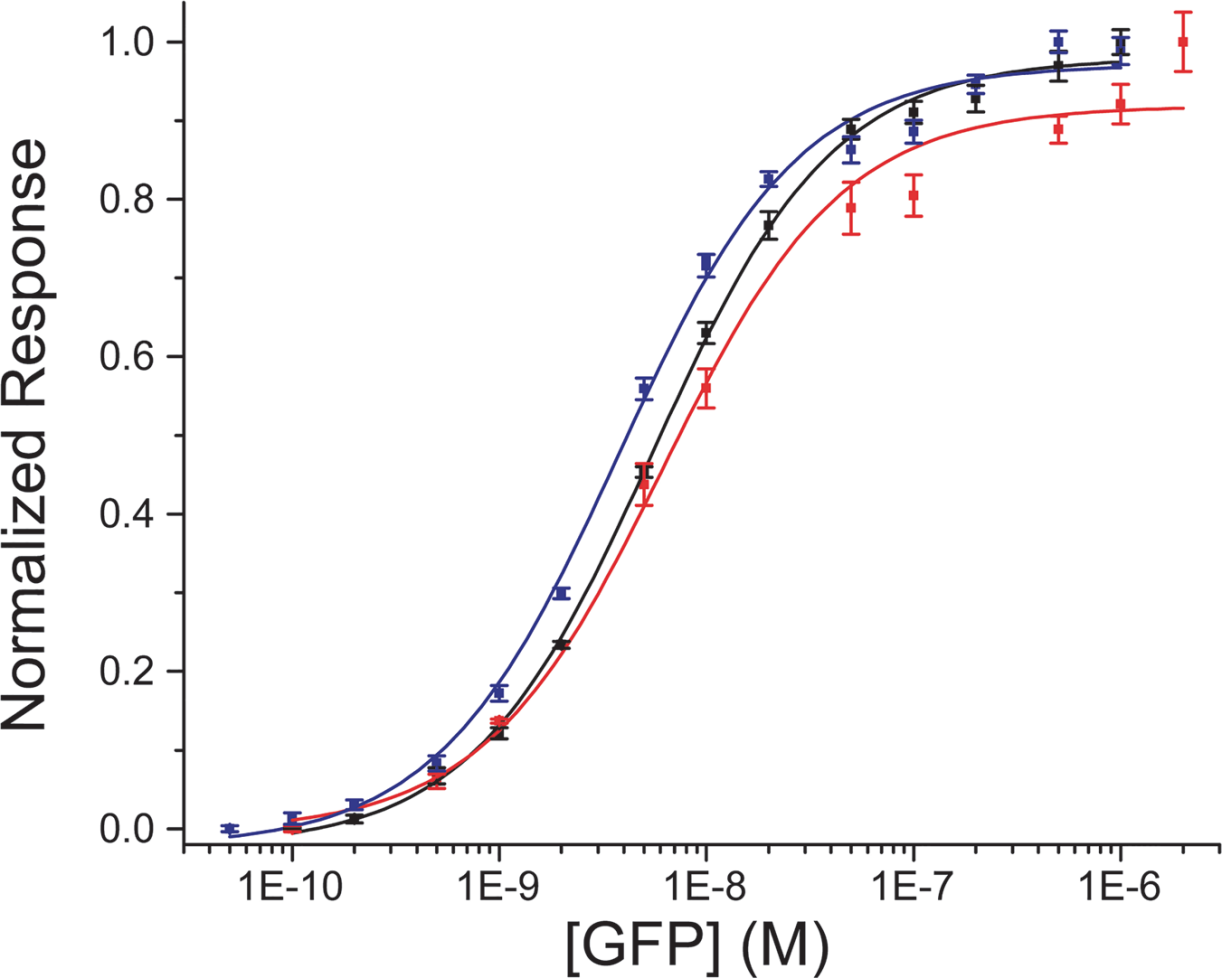
Endpoint response of the reference subtracted binding curves of the GFP:GFP-Nb interaction as function of GFP concentration. The red, blue and black squares are the raw data collected for the Ni:NTA, the anti-polyhistidine and the CAP chip respectively, and the lines are the best fit of the data set to the Hill’s equation (R^2^ = 0.99).

The capture of the GFP-Nb on the different surfaces was followed up by the study of the interaction between the nanobody and the GFP (Fig 3). The GFP-Nb was immobilized onto the different surfaces at a low density (~50 R.U.), and the high efficiency reached by the immobilization strategies (Fig 1) allowed the use of very low concentrations of GFP-Nb (less than 3 nM). GFP was observed to bind with a high affinity to the immobilized partner on all the three surfaces. Very low non-specific binding of GFP to the reference surfaces was observed (less than 1% of the specific signal measured on the activated flow cell 2). The kinetic parameters were determined by fitting the SPR data to a simple interaction model (Table 1); although the *k*_on_ of the binding complex was nearly identical on the three surfaces (*k*_on_ = (1.1 ± 0.5) x 10^6^ 1/Ms, (1.0 ± 0.4) x 10^6^ 1/Ms, (1.3 ± 0.4) x 10^6^ 1/Ms for the nanobody immobilized on Ni:NTA, anti-polyhistidine and streptavidin), the *k*_off_ was significantly higher on the Ni:NTA chip ((7.1 ± 1.1) x 10^–4^ 1/s) than on the antibody and the streptavidin functionalized surfaces ((2.9 ± 0.4) x 10^–4^ 1/s, (9.1 ± 1.3) x 10^–5^ 1/s, respectively). His-tagged ligand release from Ni:NTA surface is frequently observed [32] and this phenomenon can explain the difference in the evaluated *k*_off_. These results suggest that the streptavidin/biotin based immobilization yielded the best results in terms of ligand stability and analyte detection limit (50 pM). However, in our case the biosensor surface affected the measurements only to a minor extent. In fact, the affinity of the GFP:GFP-Nb interaction determined from a simple fitting of the end-point response to a binding isotherm revealed K_D_ values very similar for all the surfaces (Fig 4). The affinity of the interaction was determined to be slightly higher when the nanobody was immobilized on the CAP chip (K_D_ = 3.7 ± 0.3 nM) than on the anti-polyhistidine and Ni:NTA chip (K_D_ = 5.5 ± 0.5 nM and K_D_ = 6.1 ± 0.9 nM, respectively) (Table 1). These results show that, while the immobilization method can affect measurements of binding affinities to a minor extent and a stable coupling method based on streptavidin-biotin interactions is very desirable, easier-to-regenerate surfaces such as the one of the NTA chip can also be very convenient for initial experiments where a large variety of parameters usually needs to be screened.

**Table 1:** Determination of the kinetic parameters *k*_on_ and *k*_off_ and affinities K_D_ of the GFP:GFP-Nb interaction on different biosensor chip surfaces.

	***k***_on_ **(10** ^6^ **/Ms)** ^a^	***k***_off_ **(10** ^-4^ **/s)** ^a^	**K**_D_ **(nM)** ^b^
**Ni:NTA chip**	1.1 ± 0.5	7.1 ± 1.1	6.1 ± 0.9
**Anti-polyhistidine antibody CM5 chip**	1.0 ± 0.4	2.9 ± 0.4	5.5 ± 0.5
**Streptavidin on CAP chip**	1.3 ± 0.4	0.9 ± 0.1	3.7 ± 0.3

^a^ The kinetic parameters *k*_on_ and *k*_off_ were determined by fitting the SPR data to a 1:1 interaction model (Fig 3).

^b^ The K_D_ parameters of the GFP:GFP-Nb interaction were determined from a fitting of the end-point response to a binding isotherm (Fig 4).

We also noticed that the *in vitro* biotinylation of the nanobody did not affect its binding properties. This is somehow counterintuitive because the biotin groups should be randomly tethered to the free amino groups accessible on the surface of the protein resulting in different orientations of the protein on the streptavidin surface and in only a fraction of the nanobody being active for antigen recognition. On the opposite, the histidine-based immobilization methods should allow an oriented capture of the nanobody on the surface due to the presence of the histidine tag on the nanobody C-terminal end. However, in the case of the GFP-Nb, all the lysine residues lie on the surface close to the C-terminal at the antipode of the paratope (S8 Fig). This resulted in an optimal orientation of the immobilized nanobody on the SPR streptavidin sensor with its binding pocket oriented towards the solution. Furthermore the presence of the amino-space linker of the Bt-NHS minimizes steric hindrance effects of the dextran matrix and leads to a more pronounced exposure of the nanobody towards the solute enhancing its antigen-capturing properties.

The K_D_ of the GFP:GFP-Nb interaction determined by SPR investigations matched well with the results previously obtained for the same complex by interferometry (K_D_ = 1.4 nM [27]) or for similar complexes by SPR (K_D_ = 1–10 nM [14]). In order to compare the binding properties between nanobodies and classical antibodies, a biotinylated monoclonal anti-GFP antibody was also immobilized on a CAP chip and its capturing properties were tested by SPR (S9 Fig). Although similar densities of antibody and nanobody were immobilized on the surface (~4 fmol/mm^2^), the nanobody could detect lower concentration of GFP than the antibody (50 and 500 pM, respectively). Furthermore the affinity of the immobilized antibody for free GFP (K_D_ = 33 ± 5 nM, S10 Fig) was one order of magnitude lower than the one measured for the nanobody. This is probably due to the random orientation of the immobilized antibody that provided a lower accessibility of the binding pocket compared to the nanobody. Furthermore, due to avidity effects resulting from the antibodies bivalency, the sensorgrams could not be fitted by a simple interaction model, but a heterogeneous ligand model had to be used (S11 Fig). These results clearly illustrate the advantages of the nanobodies over classical antibodies as attractive partners in biosensor applications.

### GFP-Nb as recognition motif for GFP affinity tags

Having identified the properties of the interaction between the GFP-Nb and its antigen, we investigated how well it would function as a capturing molecule in SPR assays. The GFP-Nb diluted in sodium acetate buffers with pH varying between 4.0 and 6.0 was injected onto the surface of a CM5 chip (S12 Fig). The optimum conditions for immobilization of the protein were obtained using a solution of pH 5.4 that is more neutral than the one that needs to be used to immobilize anti-GFP antibody on a CM5 surface (S13 Fig). The GFP-Nb was then immobilized on the surface of the CM5 chip by covalent coupling of the free amino groups of the protein surface and the activated carboxylic groups of the CM5 dextran matrix. The amount of nanobody immobilized onto the CM5 surface (~2500 R.U.) corresponded to ~200 fmol/mm^2^ that is more than three times the amount of anti-GFP antibody immobilized in a similar fashion on a CM5 chip (~10000 R.U. corresponding to ~65 fmol/mm^2^).

**Fig 5:**
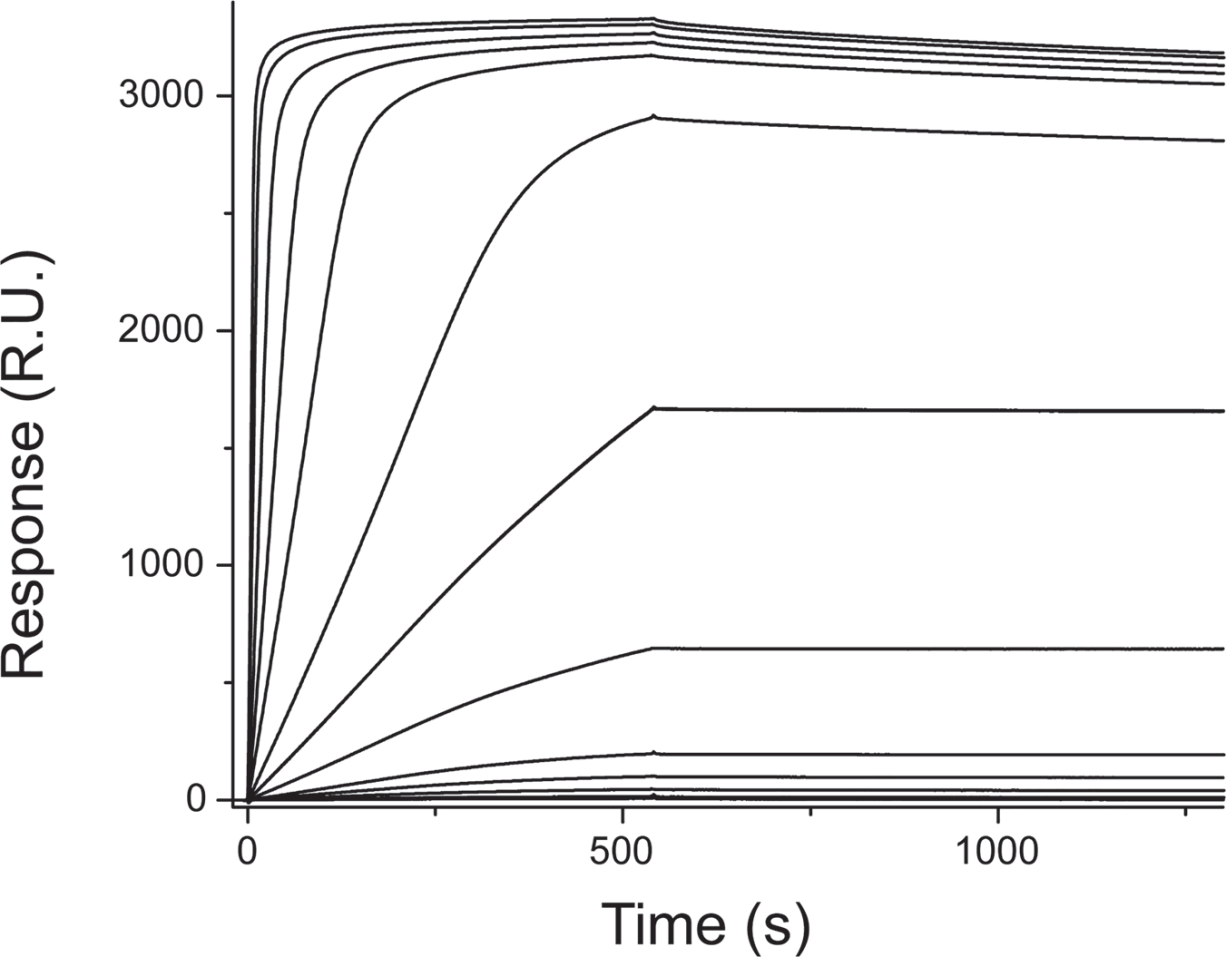
Interaction of GFP with a CM5 surface functionalized with GFP-Nb. Depicted sensorgrams were obtained for GFP-Nb concentration of 0.2, 0.5, 1, 2, 5, 7, 10, 15, 20, 50, 100, 200, 500, 1000 nM (from bottom to top curve).

Different concentrations of GFP were injected on the chip functionalized with the nanobody (Fig 5); the maximum response achieved was 3400 R.U. that is ~75% of the theoretical maximum binding capacity of the functionalized surface for GFP. As for the streptavidin-mediated immobilization, the presence of the lysine residues at the antipode of the binding sites leads to a *quasi*-site-specific and to a highly active immobilization of the probe. On the opposite, the maximum amount of GFP that could be immobilized on the anti-GFP antibody functionalized surface was ~700 R.U. that is only 21% of the evaluated theoretical value (S14 Fig). This value is almost five times lower than the maximum binding capacity of the CM5 chip coupled with GFP-Nb indicating that steric hindrance effects due to the large size of the antibodies, their random immobilization and the possible loss of functionality during the coupling procedure affect strongly their binding properties. In addition the GFP-Nb bound to the CM5 chip shows a higher affinity for the GFP (K_D_ = 9.6 ± 0.2 nM, S15 Fig) than the anti-GFP antibody coupled to the same matrix (K_D_ = 30.0 ± 0.2 nM, S16 Fig).

As these results indicated that the GFP-Nb could be a valid, superior alternative to large, bivalent anti-GFP antibodies as capturing molecules for GFP fusion proteins we also investigated the stability of the immobilized nanobody. We first followed the dissociation of the GFP from the capturing agent for 10 hs (S17 Fig). The results indicated a very low off rate (*k*_off_ = 1.4 x 10^–5^ s^-1^) with less than 10% of the GFP being removed from the surface after 10 hs. Multiple cycles of GFP injection and surface regeneration indicated that the GFP-Nb was strongly immobilized onto the CM5 surface and that the functionalized chip could be re-used without any significant loss or variation in binding signal (S18 Fig). Furthermore the regeneration of the GFP-Nb-coupled CM5 surface with 10 mM glycine pH 2.0 removed less than 0.1% of immobilized proteins per cycle. As efficient regeneration of the surface is fundamental for the development of a capturing agent, several regeneration conditions were also tested. Results indicated that only 10 mM glycine-HCl (pH 1 or 2) removed completely the GFP from the surface. 50 mM NaOH solution could partially regenerate the surface, while other commonly used regeneration solutions (5 M NaCl, 4 M MgCl_2_, 0.5% SDS and 50% ethylene glycol) would not affect the GFP-Nb:GFP complex (S19 Fig). This result is particularly interesting for interaction studies between GFP fusion proteins and their binding partners. Conventional surface-based studies require relatively large amounts of both fusion proteins and partners because following every binding event the biosensor surface is regenerated by removing both the proteins. On the opposite, in the case of GFP-tagged proteins immobilized on the GFP-Nb surface, only the interaction partners could be removed by using one of the solutions listed above leaving the GFP fusion protein free to interact with a new partner.

### GFP-Nb stability

Many applications of single domain antibodies in biosensing technologies will eventually depend on their stability and on the retention of their antigen-binding capacity in different conditions (*i*.*e*. extreme pH, temperature and ionic strength, presence of solvents and/or detergents…). Furthermore the surface-immobilized nanobodies need to be robust and resist to multiple regenerations conditions in order to reduce the overall cost of the biosensors. Previous experiments have demonstrated that nanobodies retain most of their properties when exposed to harsh conditions in solution [12,13,14,15,33]. Here we probed the antigen-binding properties of the nanobodies under three extremely different denaturing conditions, using temperature, chemicals and salt.

**Fig 6:**
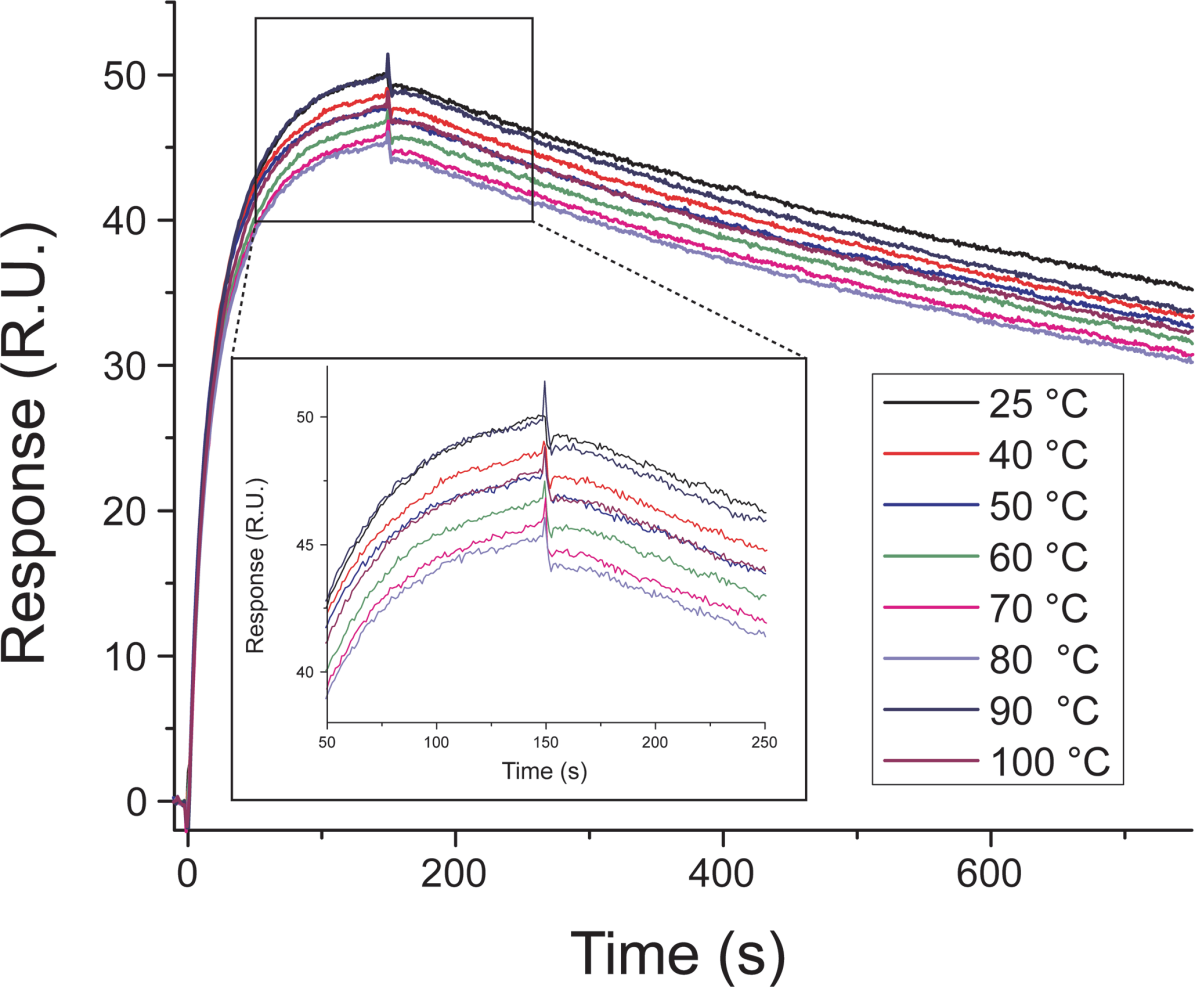
Effect of temperature on GFP-Nb stability. The sensorgrams illustrates the binding of 30 nM GFP to the GFP-Nb immobilized on a Ni:NTA surface. Before immobilization, the nanobody had been heated at the temperatures indicated in the figure for 30 min and then allowed to cool down at room temperature for 30 min. The inset shows a zoom of the sensorgrams recorded between 50 s and 250 s.

We started by examining the thermal stability of the GFP-Nb by monitoring its interaction with GFP by SPR after being heated for 30 min and cooled at room temperature. Fig 6 shows the sensorgrams recorded for the GFP:GFP-Nb interaction with the nanobody being incubated at several temperatures (range 25°C—100°C) before immobilization on the Ni:NTA surface. The thermal shock did not affect the nanobody ability to bind to the surface nor its interaction with the GFP (R_max_ = 47.6 ± 1.5); the evaluated affinities were identical within the error for all the temperature range (*k*_on_ = (1.5 ± 0.1) x 10^6^ 1/Ms, *k*_off_ = (6.3 ± 0.2) x 10^–4^ 1/s). These results demonstrate that the single domain antibodies fully recover their biological activity after a complete thermal unfolding/refolding cycle even at temperatures higher than the melting temperature (T_m_ = 60–80°C [14]). This is particularly interesting when compared to the poor thermo-stability of classical antibodies for which incubation at temperature higher than their T_m_ often results in exposure of inner hydrophobic pockets and in their aggregation and precipitation.

The exceptional stability of the GFP-Nb was also evident from pH-induced unfolding experiments. The protein samples, incubated overnight in NTA running buffer of pH values varying between 4.0 and 10.5 and then immobilized on a Ni:NTA surface, were tested for their ability to bind the antigen by SPR (S20 Fig). The nanobody was found to bind to the Ni:NTA ~50% less at pH < 6 (S21 Fig). This is probably due to repulsion effects between the negatively charged surface of the biosensor (pKa = 3.5) and some negative residues exposed on the protein surface. However, upon immobilization, the nanobody retained its antigen-binding capacity with association and dissociation constants very homogeneous over the all pH range (*k*_on_ = (1.4 ± 0.2) x 10^6^ 1/Ms, *k*_off_ = (6.4 ± 0.8) x 10^–4^ 1/s).

Further experiments examined the effect of the buffer ionic strength on the antigen-nanobody binding kinetics. SPR measurements demonstrated that incubating GFP with high concentrations of NaCl (up to 1 M) did not impair the nanobody ability to recognize its binding partner (S22 Fig). The association and dissociation constants appear to be independent from the NaCl concentration (*k*_on_ = (1.5 ± 0.2) x 10^6^ 1/Ms, *k*_off_ = (6.3 ± 0.5) x 10^–4^ 1/s). These results seem to be a further indication that only hydrophobic interaction at the binding interface between the GFP-Nb and the GFP are responsible of the complex high affinity (residue Phe102 of GFP-Nb interacts with Leu221, Ala206, and Phe223 of GFP and Trp47 of GFP-Nb interacts with Val176 and Ser175 of GFP) [27].

Our experiments demonstrate once more the remarkable stability and refolding properties of the single domain antibodies that are particularly important in biosensor applications where proteins need to be chemically modified to be attached to a solid support.

## Conclusions

Over the last years several studies have demonstrated the use of single domain antibodies for biotechnological applications. These proteins can be easily expressed in several hosts, are small and more stable than conventional antibodies, have high solubility properties and high specificity and selectivity for their interaction partners. Here, we have investigated the properties of a nanobody against GFP by using SPR; the achieved results are not limited to SPR applications, but they can be easily applied to any other biosensing platform.

Robust immobilization of the nanobody can be successfully achieved on Ni:NTA and anti-polyhistidine chips *via* his-tag, on streptavidin-functionalized surfaces *via* biotin tag or directly on carboxylic-terminated surfaces by chemical coupling. We evaluate and compare the advantages and the limitations of all these immobilization schemes to detect specific antigens; and show that nanobodies outperform large classical antibodies in terms of both binding capacity and affinities. Thanks to the presence of lysine residues at the antipode of the binding site, the GFP-Nb can be immobilized in an oriented manner on the biosensor surface allowing for an optimal configuration for efficient capture of the antigen of interest. Furthermore the GFP-Nb shows a high stability; it can be strongly immobilized on the biosensor surfaces and resist a wide variety of extreme conditions such as high temperature, solution of low or high pH and high salt content.

Our results demonstrate that the GFP-Nb combines a series of favorable properties compatible with the development of robust capturing tools with high sensitivity, selectivity and specificity. Any wild-type GFP, eGFP as well as YFP and eYFP-fusion proteins could be immobilized on a surface functionalized with the GFP-Nb and the interaction with the binding partner evaluated. Furthermore the availability and the development of novel nanobodies against other affinity tags (*i*.*e*. GST, RFP, short peptides…) paves the way to a wide variety of applications both in biosensing and diagnostics.

## Supporting Information

S1 Fig Reproducibility of the GFP-Nb immobilization. The sensorgrams show five runs of immobilization of the GFP-Nb (35 nM) on the Ni:NTA surface and the replicates indicate the reproducibility of the assay. The inset shows a zoom of the sensorgrams recorded between 150 s and 400 s.
(PNG)

S2 Fig Binding of GFP-Nb to SPR biosensor surfaces. The SPR sensorgrams show the interaction between the GFP-Nb and a NTA surface activated with Ni ions (top), and a CM5 chip functionalized with anti-polyhistidine antibodies (bottom). Black lines are raw data and the red lines are the fitting to a 1:1 binding model. Depicted sensorgrams were obtained for GFP-Nb concentration of 50, 100, 250, 500, 1000, 1500 nM (from bottom to top curve).
(TIF)

S3 Fig Endpoint response of the reference subtracted binding curves of the GFP-Nb/surfaces interaction as function of GFP-Nb concentration. The red, blue and black squares are the raw data for the Ni:NTA, the anti-polyhistidine and the CAP chip respectively, and the lines are the best fit of the data set to the Hill’s equation (Hill’s coefficient set equal to 1).
(PNG)

S4 Fig Binding of his-eGFP to Ni:NTA SPR biosensor surfaces. The SPR sensorgrams show the interaction between his-eGFP and a NTA surface activated with Ni^2+^ ions. Depicted sensorgrams were obtained for GFP-Nb concentration of 5, 10, 35, 50, 100, 250, 500, 1000 nM (from bottom to top curve).
(PNG)

S5 Fig Binding of his-eGFP to a CM5 chip functionalized with anti-polyhistidine antibodies. Depicted sensorgrams were obtained for GFP-Nb concentration of 1, 5, 10, 35, 50, 100, 250, 500, 1000 nM (from bottom to top curve).
(PNG)

S6 Fig Anti-polyhistidine:GFP-Nb interaction. The normalized SPR sensorgrams show the interaction between a CM5 chip functionalized with anti-polyhistidine antibodies and the GFP-Nb (black line) and the his-eGFP (red line). Depicted sensorgrams were obtained for protein concentration of 35 nM.
(PNG)

S7 Fig Structure of GFP-Nb. The protein ribbon structure is colored in red with the C-terminal-where the poly-histidine tag is inserted—shown in blue (PDB, 3OGO).
(TIF)

S8 Fig Structure of the GFP:GFP-Nb complex. GFP ribbon structure is colored in green, the GFP-Nb is colored in red with the lysine residues highlighted as yellow spheres and the C-terminal highlighted as blue spheres (PDB, 3OGO).
(TIF)

S9 Fig GFP:anti-GFP complex. Binding of GFP to anti-GFP antibody immobilized on streptavidin on CAP chip. Depicted sensorgrams were obtained for GFP concentration of 0.1, 0.5, 1, 5, 10, 50, 100, 200, 500, 1000 nM (from bottom to top curve).
(PNG)

S10 Fig Endpoint response of the reference subtracted binding curves of the GFP:anti-GFP antibody interaction as function of GFP concentration. The squares are the raw data and the line is the best fit of the data set to the Hill’s equation (Hill’s coefficient set equal to 1).
(PNG)

S11 Fig GFP:anti-GFP complex. Binding of GFP to anti-GFP antibody immobilized on streptavidin on CAP chip. Black lines are raw data and the red lines are the fitting to a 1:1 binding model. Depicted sensorgrams were obtained for GFP concentration of 0.1, 0.5, 1, 5, 10, 50, 100, 200, 500, 1000 nM.
(PNG)

S12 Fig GFP-Nb pre-concentration scouting results. The protein was diluted in 10 mM sodium acetate buffer with pH varying between 4.0 and 6.0. The binding increases as the pH increases from 4.0 to 5.4. At pH higher than 5.7 the sensorgrams shows some dissociation after the initial binding. The bound nanobody dissociated completely from the surface at the end of the injection independently from the solution tested. The optimum pH for protein immobilization is 5.4.
(PNG)

S13 Fig Anti-GFP antibody pre-concentration scouting results. The protein was diluted in 10 mM sodium acetate buffer with pH varying between 4.0 and 6.0. The maximum binding is achieved at pH values between 4.3 and 4.6; the binding decreases for pH values higher than 4.6. The bound antibody dissociated completely from the surface at the end of the injection independently from the solution tested. The optimum pH for protein immobilization is 4.6.
(PNG)

S14 Fig Interaction of GFP with a CM5 surface functionalized with anti-GFP antibody. Depicted sensorgrams were obtained for GFP-Nb concentration of 0.2, 0.5, 1, 2, 5, 7, 10, 15, 20, 50, 100, 200, 500, 1000 nM (from bottom to top curve).
(PNG)

S15 Fig Endpoint response of the reference subtracted binding curves of the GFP:GFP-Nb interaction as function of GFP concentration. GFP-Nb was immobilized onto a CM5 chip via amino-coupling. The squares are the raw data and the line is the best fit of the data set to the Hill’s equation (Hill’s coefficient set equal to 1).
(PNG)

S16 Fig Endpoint response of the reference subtracted binding curves of the GFP:anti-GFP antibody interaction as function of GFP concentration. Anti-GFP was immobilized onto a CM5 chip via amino-coupling. The squares are the raw data and the line is the best fit of the data set to the Hill’s equation (Hill’s coefficient set equal to 1).
(PNG)

S17 Fig Stability of the GFP:GFP-Nb interaction. The sensorgrams show a run of immobilization of the GFP (10 nM) on the CM5 surface functionalized with the GFP-Nb. Dissociation of the GFP was followed for 36000 s. The black curve is the raw data and the red line is the fitting to a 1:1 binding model.
(PNG)

S18 Fig Reproducibility of the GFP:GFP-Nb interaction. The sensorgrams show 50 runs of immobilization of the GFP (10 nM) on the CM5 surface functionalized with the GFP-Nb. The inset shows the R_max_ value as a function of the cycle number.
(PNG)

S19 Fig Effect of regeneration solutions on GFP:GFP-Nb interaction. The sensorgrams show the immobilization of 10 nM GFP on a CM5 surface functionalized with the GFP-Nb followed by regeneration with a series of solutions. While 10 mM glycine solution removed completely the GFP from the surface and 50 mM NaOH removed partially the GFP, all the other solutions did not affect the binding of GFP to the immobilized nanobody.
(PNG)

S20 Fig Effect of pH on GFP-Nb stability. The sensorgrams illustrates the binding of 30 nM GFP to the GFP-Nb immobilized on a Ni:NTA surface. Before immobilization, the nanobody was diluted in NTA running buffer of several pH (range 4.0–10.5, see legend) and then applied to the Ni:NTA surface.
(PNG)

S21 Fig Effect of pH on GFP-Nb binding to Ni:NTA surface. The plot shows the maximum response of the Ni:NTA chip for the nanobody as a function of the solution pH.
(PNG)

S22 Fig Effect of ionic strength on GFP-Nb stability. The sensorgrams illustrates the binding of the GFP-nanobody immobilized on a Ni:NTA surface to 30 nM GFP diluted in NTA running buffer with different concentration of NaCl (range 0.15 M-1.0 M, see legend).
(PNG)

## References

[1] Hamers-Casterman C, Atarhouch T, Muyldermans S, Robinson G, Hammers C, Songa EB, et al (1993) Naturally occurring antibodies devoid of light chains. Nature 363: 446–448. 8502296 10.1038/363446a0

[2] Huang L, Muyldermans S, Saerens D (2010) Nanobodies: proficient tools in diagnostics. Expert Review of Molecular Diagnostics 10: 777–785. 10.1586/erm.10.62 20843201

[3] Kirchhofer A, Helma J, Schmidthals K, Frauer C, Cui S, Karcher A, et al (2010) Modulation of protein properties in living cells using nanobodies. Nature structural & molecular biology 17: 133–138. 10.1038/nsmb.1727 20010839

[4] Muyldermans S (2013) Nanobodies: natural single-domain antibodies. Annual review of biochemistry 82: 775–797. 10.1146/annurev-biochem-063011-092449 23495938

[5] Pardon E, Laeremans T, Triest S, Rasmussen SGF, Wohlkonig A, Ruf A, et al (2014) A general protocol for the generation of nanobodies for structural biology. Nature protocols 9: 674–693. 10.1038/nprot.2014.039 24577359 PMC4297639

[6] Korotkov KV, Pardon E, Steyaert J, Hol WG (2009) Crystal structure of the N-terminal domain of the secretin GspD from ETEC determined with the assistance of a nanobody. Structure 17: 255–265. 10.1016/j.str.2008.11.011 19217396 PMC2662362

[7] Broisat A, Hernot S, Toczek J, De Vos J, Riou LM, Martin S, et al (2012) Nanobodies targeting mouse/human VCAM1 for the nuclear imaging of atherosclerotic lesions. Circulation research 110: 927–937. 10.1161/CIRCRESAHA.112.265140 22461363 PMC3918224

[8] Kierny MR, Cunningham TD, Kay BK (2012) Detection of biomarkers using recombinant antibodies coupled to nanostructured platforms. Nano reviews 3 10.3402/nano.v3i0.18496 22833780 PMC3404449

[9] Van de Broek B, Devoogdt N, D'Hollander A, Gijs H-L, Jans K, Lagae L, et al (2011) Specific cell targeting with nanobody conjugated branched gold nanoparticles for photothermal therapy. ACS nano 5: 4319–4328. 10.1021/nn1023363 21609027

[10] Holz J-B (2012) The TITAN trial—assessing the efficacy and safety of an anti-von Willebrand factor nanobody in patients with acquired thrombotic thrombocytopenic purpura. Transfusion and Apheresis Science 46: 343–346. 10.1016/j.transci.2012.03.027 22475545

[11] Muyldermans S, Baral T, Retamozzo VC, De Baetselier P, De Genst E, Kinne J, et al (2009) Camelid immunoglobulins and nanobody technology. Veterinary immunology and immunopathology 128: 178–183. 10.1016/j.vetimm.2008.10.299 19026455

[12] Dolk E, van Vliet C, Perez JM, Vriend G, Darbon H, Ferrat G, et al (2005) Induced refolding of a temperature denatured llama heavy-chain antibody fragment by its antigen. PROTEINS: Structure, Function, and Bioinformatics 59: 555–564. 15778955 10.1002/prot.20378

[13] Van der Linden R, Frenken L, De Geus B, Harmsen M, Ruuls R, Stok W, et al (1999) Comparison of physical chemical properties of llama V_HH_ antibody fragments and mouse monoclonal antibodies. Biochimica et Biophysica Acta (BBA)-Protein Structure and Molecular Enzymology 1431: 37–46. 10209277 10.1016/s0167-4838(99)00030-8

[14] Dumoulin M, Conrath K, Van Meirhaeghe A, Meersman F, Heremans K, Frenken LG, et al (2002) Single-domain antibody fragments with high conformational stability. Protein Science 11: 500–515. 11847273 10.1110/ps.34602 PMC2373476

[15] Dolk E, Van Der Vaart M, Hulsik DL, Vriend G, de Haard H, Spinelli S, et al (2005) Isolation of llama antibody fragments for prevention of dandruff by phage display in shampoo. Applied and environmental microbiology 71: 442–450. 15640220 10.1128/AEM.71.1.442-450.2005 PMC544197

[16] Roach P, Farrar D, Perry CC (2006) Surface tailoring for controlled protein adsorption: effect of topography at the nanometer scale and chemistry. Journal of the American Chemical Society 128: 3939–3945. 16551101 10.1021/ja056278e

[17] Anderson GP, Bernstein RD, Swain MD, Zabetakis D, Goldman ER (2010) Binding kinetics of antiricin single domain antibodies and improved detection using a B chain specific binder. Analytical chemistry 82: 7202–7207. 10.1021/ac100961x 20687583

[18] Anderson GP, Legler PM, Zabetakis D, Goldman ER (2012) Comparison of immunoreactivity of staphylococcal enterotoxin b mutants for use as toxin surrogates. Analytical chemistry 84: 5198–5203. 10.1021/ac300864j 22681495

[19] Conrath K, Pereira AS, Martins CE, Timoteo CG, Tavares P, Spinelli S, et al (2009) Camelid nanobodies raised against an integral membrane enzyme, nitric oxide reductase. Protein Science 18: 619–628. 10.1002/pro.69 19241371 PMC2760367

[20] Saerens D, Frederix F, Reekmans G, Conrath K, Jans K, Brys L, et al (2005) Engineering camel single-domain antibodies and immobilization chemistry for human prostate-specific antigen sensing. Analytical chemistry 77: 7547–7555. 16316161 10.1021/ac051092j

[21] Huang L, Reekmans G, Saerens D, Friedt J-M, Frederix F, Francis L, et al (2005) Prostate-specific antigen immunosensing based on mixed self-assembled monolayers, camel antibodies and colloidal gold enhanced sandwich assays. Biosensors and bioelectronics 21: 483–490. 16076438 10.1016/j.bios.2004.11.016

[22] Schornack S, Fuchs R, Huitema E, Rothbauer U, Lipka V, Kamoun S (2009) Protein mislocalization in plant cells using a GFP-binding chromobody. The Plant Journal 60: 744–754. 10.1111/j.1365-313X.2009.03982.x 19686537

[23] Rothbauer U, Zolghadr K, Muyldermans S, Schepers A, Cardoso MC, Leonhardt H (2008) A versatile nanotrap for biochemical and functional studies with fluorescent fusion proteins. Molecular & Cellular Proteomics 7: 282–289. 17951627 10.1074/mcp.M700342-MCP200

[24] Rothbauer U, Zolghadr K, Tillib S, Nowak D, Schermelleh L, Gahl A, et al (2006) Targeting and tracing antigens in live cells with fluorescent nanobodies. Nature methods 3: 887–889. 17060912 10.1038/nmeth953

[25] Drew D, Lerch M, Kunji E, Slotboom D-J, de Gier J-W (2006) Optimization of membrane protein overexpression and purification using GFP fusions. Nature methods 3: 303–313. 16554836 10.1038/nmeth0406-303

[26] Pichler G, Jack A, Wolf P, Hake SB (2012) Versatile toolbox for high throughput biochemical and functional studies with fluorescent fusion proteins. PloS one 7: e36967 10.1371/journal.pone.0036967 22606318 PMC3350483

[27] Kubala MH, Kovtun O, Alexandrov K, Collins BM (2010) Structural and thermodynamic analysis of the GFP:GFP-nanobody complex. Protein Science 19: 2389–2401. 10.1002/pro.519 20945358 PMC3009406

[28] Vick JE, Johnson ET, Choudhary S, Bloch SE, Lopez-Gallego F, Srivastava P, et al (2011) Optimized compatible set of BioBrick vectors for metabolic pathway engineering. Applied microbiology and biotechnology 92: 1275–1286. 10.1007/s00253-011-3633-4 22033566

[29] Cormack BP, Valdivia RH, Falkow S (1996) FACS-optimized mutants of the green fluorescent protein (GFP). Gene 173: 33–38. 8707053 10.1016/0378-1119(95)00685-0

[30] Patterson GH, Knobel SM, Sharif WD, Kain SR, Piston DW (1997) Use of the green fluorescent protein and its mutants in quantitative fluorescence microscopy. Biophysical journal 73: 2782–2790. 9370472 10.1016/S0006-3495(97)78307-3 PMC1181180

[31] Della Pia EA, Holm JV, Lloret N, Le Bon C, Popot J-L, Zoonens M, et al (2014) A step closer to membrane protein multiplexed nanoarrays using biotin-doped polypyrrole. ACS nano 8: 1844–1853. 10.1021/nn406252h 24476392 PMC4004317

[32] Khan F, He M, Taussig MJ (2006) Double-hexahistidine tag with high-affinity binding for protein immobilization, purification, and detection on ni-nitrilotriacetic acid surfaces. Analytical chemistry 78: 3072–3079. 16642995 10.1021/ac060184l

[33] Dejaegere A, Choulier L, Lafont V, De Genst E, Altschuh Dl (2005) Variations in antigen-antibody association kinetics as a function of pH and salt concentration: a QSAR and molecular modeling study. Biochemistry 44: 14409–14418. 16262241 10.1021/bi050986v

