# Supplementary figures and images for: Single Domain Antibodies as a Powerful Tool for High Quality Surface Plasmon Resonance Studies
Source: PLoS One. 2015 Mar 30;10(3):e0124303. doi: 10.1371/journal.pone.0124303 (PMC4378939; doi:10.1371/journal.pone.0124303)

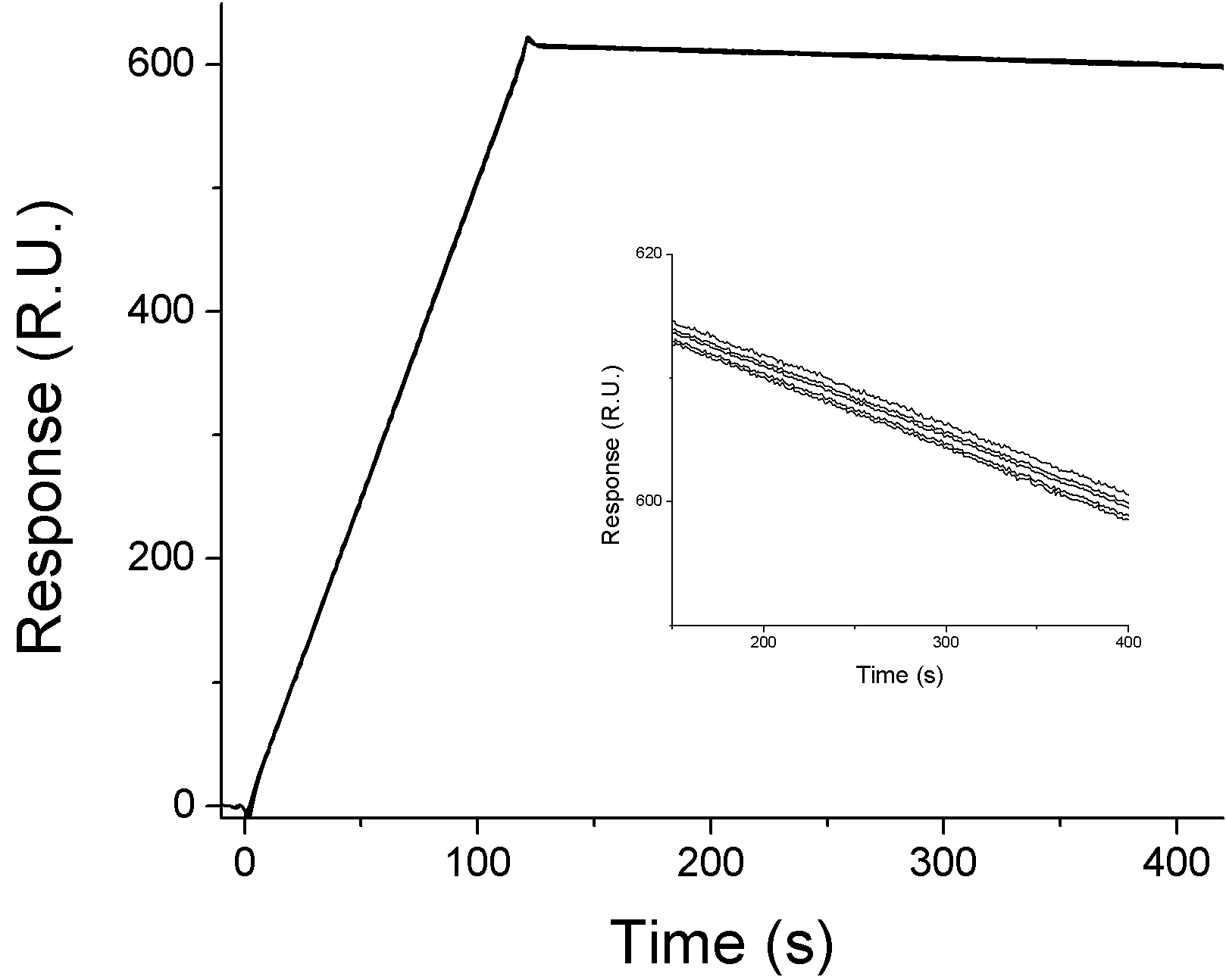

Supplement: S1 Fig — The sensorgrams show five runs of immobilization of the GFP-Nb (35 nM) on the Ni:NTA surface and the replicates indicate the reproducibility of the assay. The inset shows a zoom of the sensorgrams recorded between 150 s and 400 s. (PNG) [file pone.0124303.s001.png]

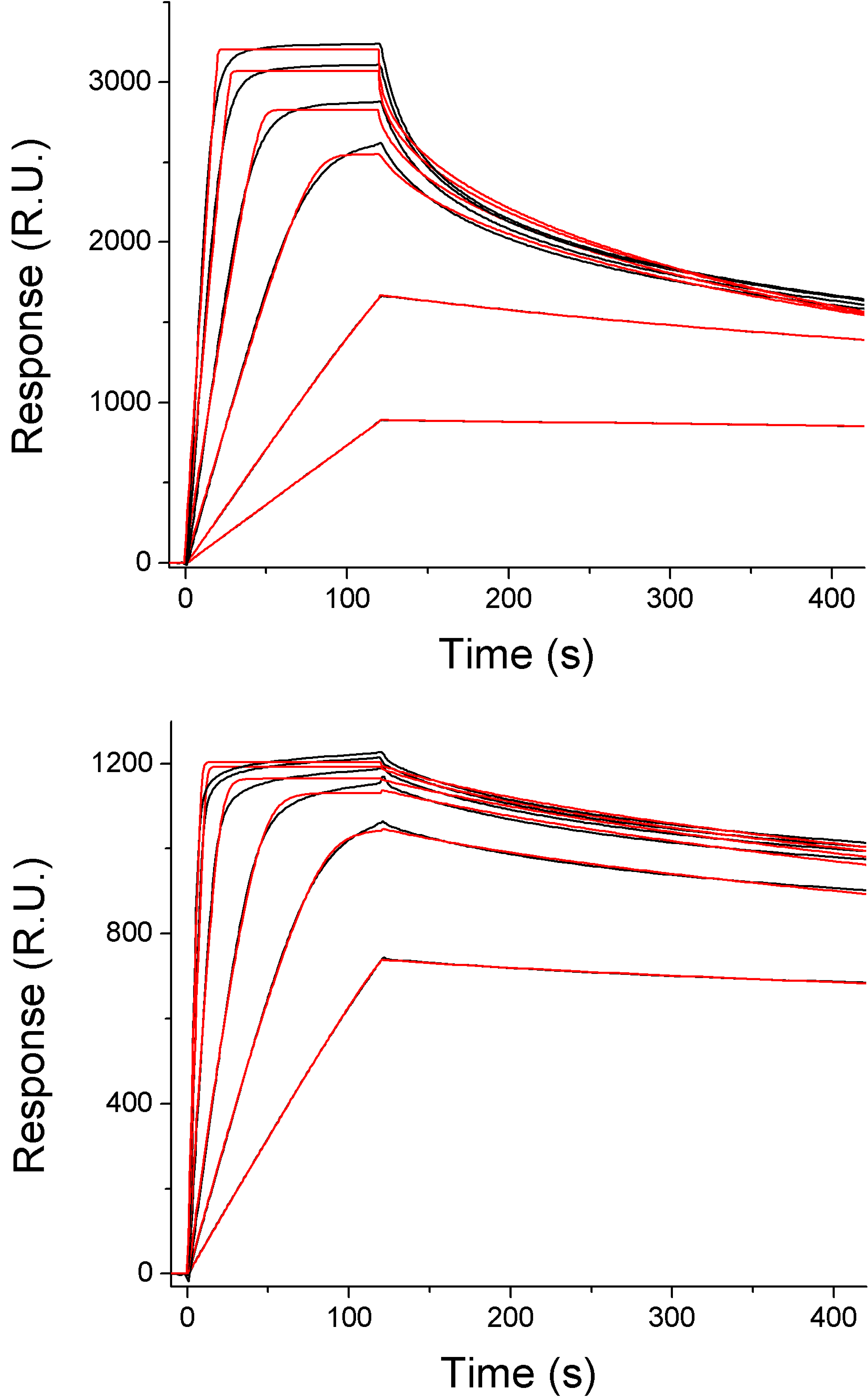

Supplement: S2 Fig — The SPR sensorgrams show the interaction between the GFP-Nb and a NTA surface activated with Ni ions (top), and a CM5 chip functionalized with anti-polyhistidine antibodies (bottom). Black lines are raw data and the red lines are the fitting to a 1:1 binding model. Depicted sensorgrams were obtained for GFP-Nb concentration of 50, 100, 250, 500, 1000, 1500 nM (from bottom to top curve). (TIF) [file pone.0124303.s002.tif]

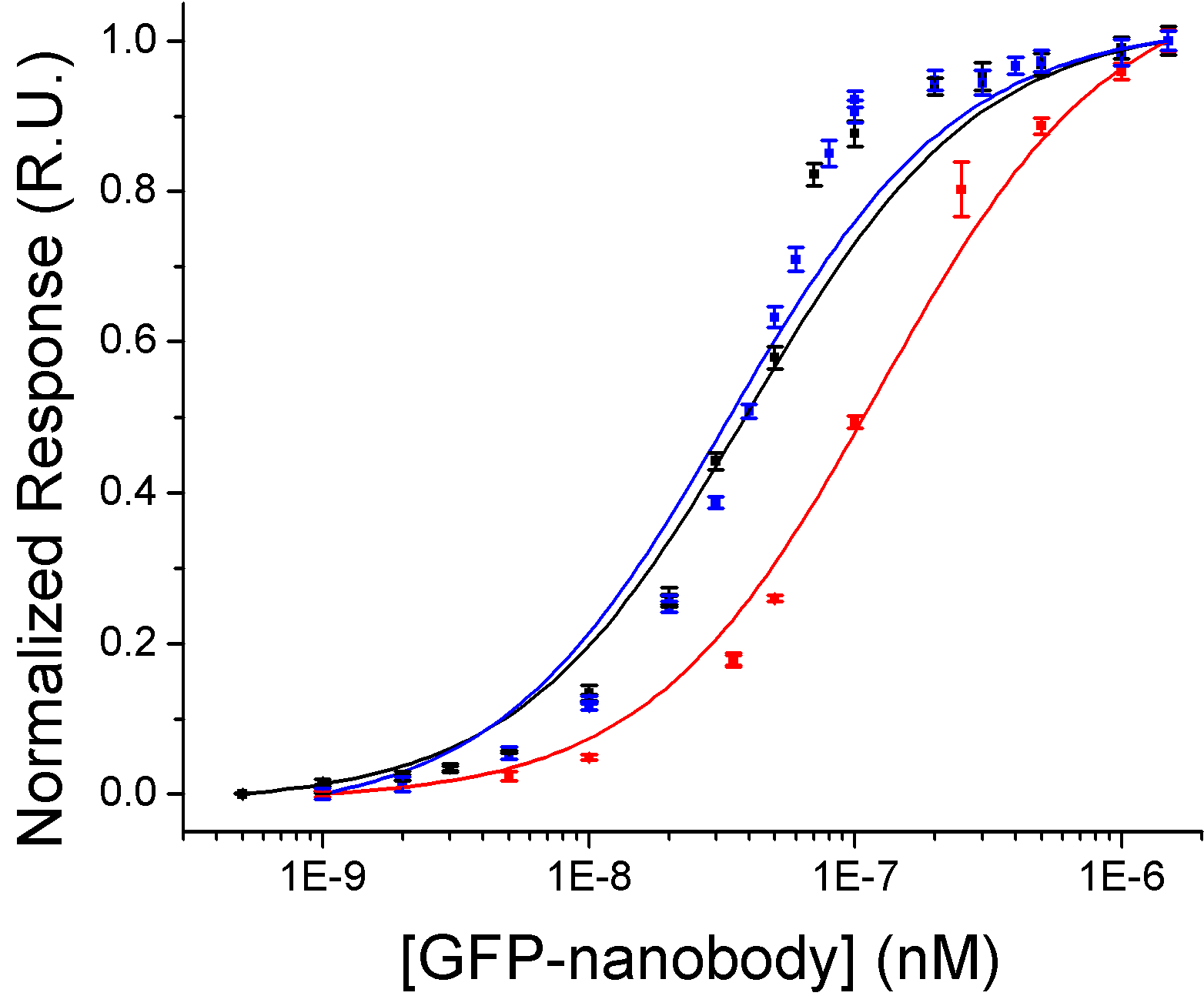

Supplement: S3 Fig — The red, blue and black squares are the raw data for the Ni:NTA, the anti-polyhistidine and the CAP chip respectively, and the lines are the best fit of the data set to the Hill’s equation (Hill’s coefficient set equal to 1). (PNG) [file pone.0124303.s003.png]

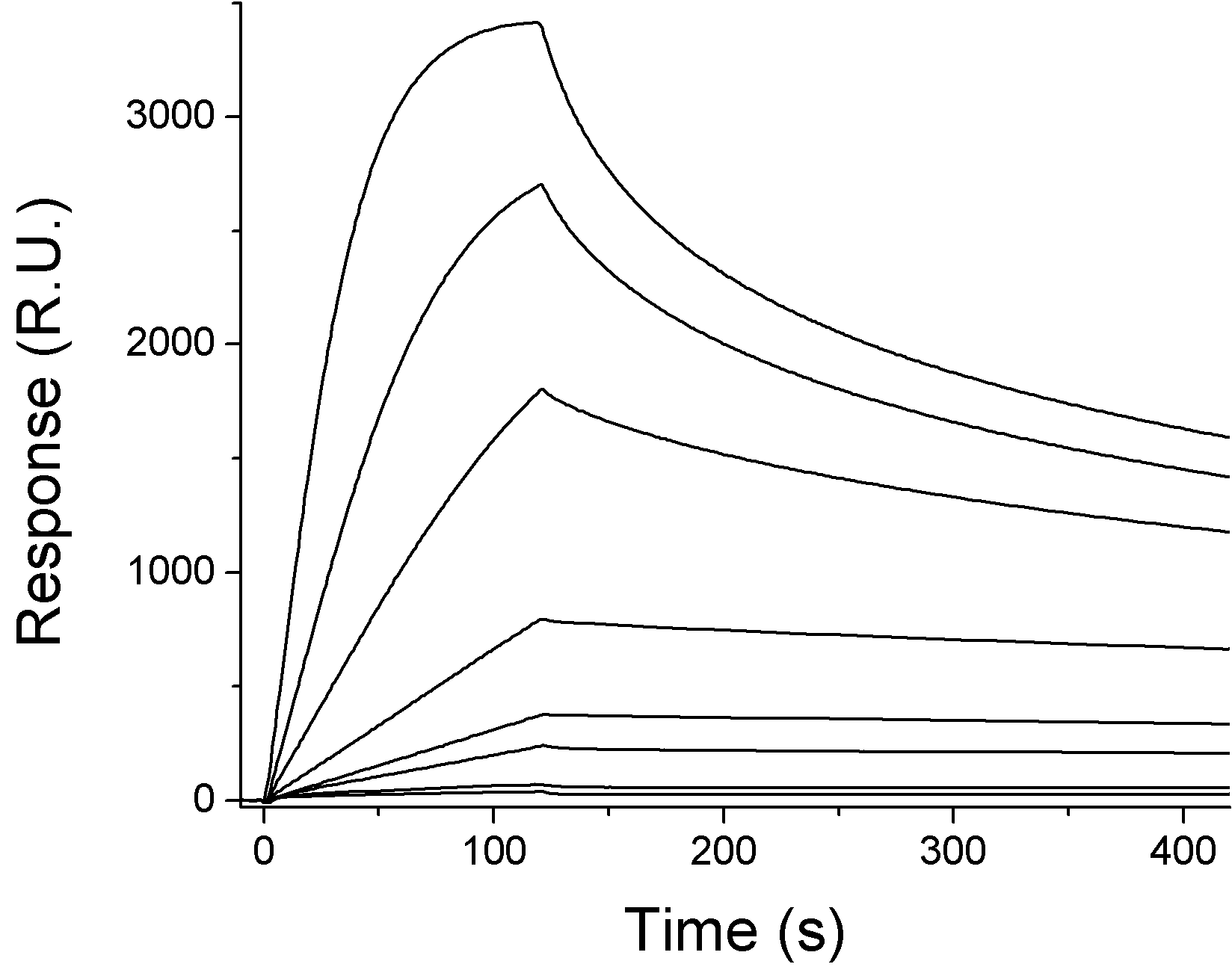

Supplement: S4 Fig — The SPR sensorgrams show the interaction between his-eGFP and a NTA surface activated with Ni2+ ions. Depicted sensorgrams were obtained for GFP-Nb concentration of 5, 10, 35, 50, 100, 250, 500, 1000 nM (from bottom to top curve). (PNG) [file pone.0124303.s004.png]

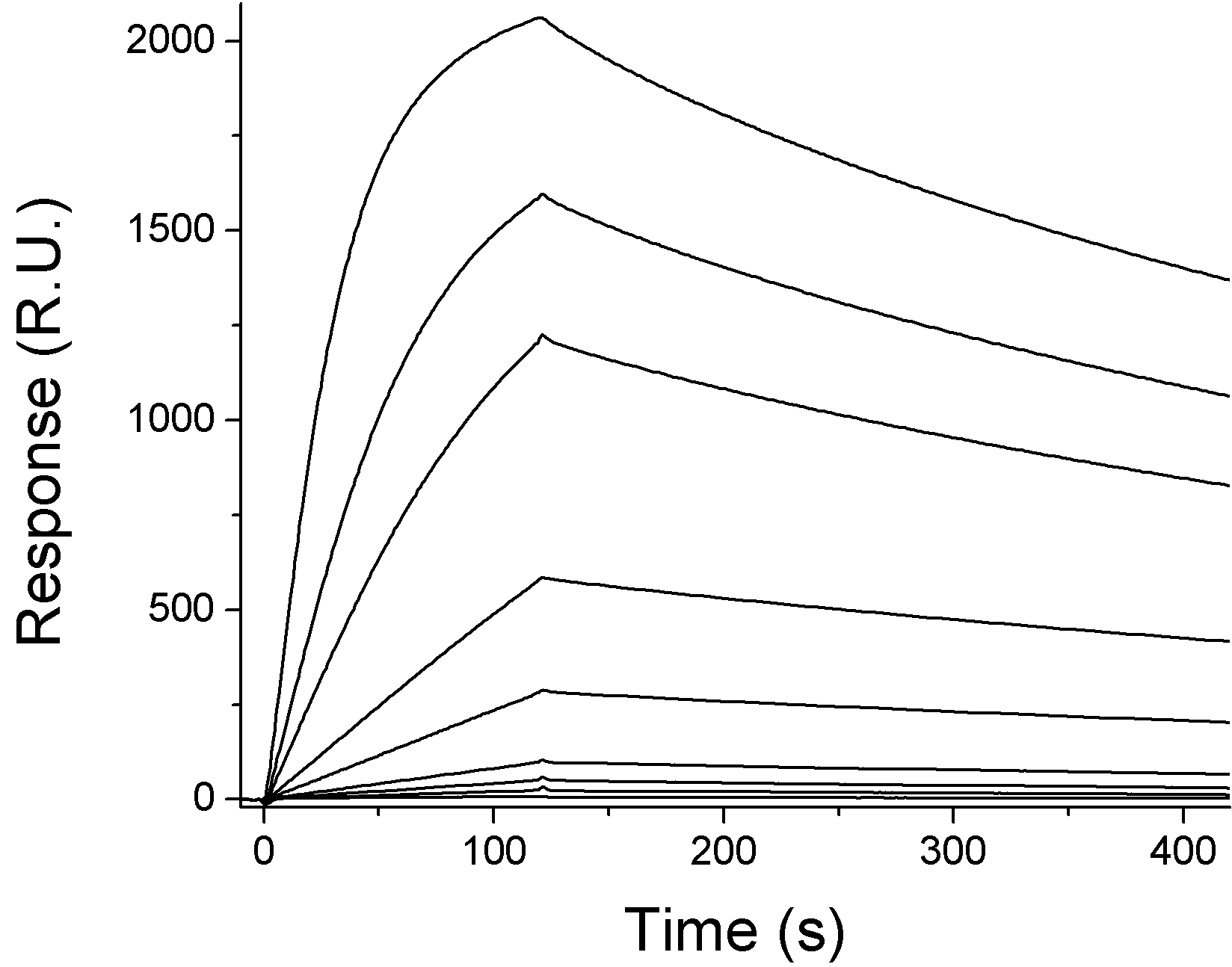

Supplement: S5 Fig — Depicted sensorgrams were obtained for GFP-Nb concentration of 1, 5, 10, 35, 50, 100, 250, 500, 1000 nM (from bottom to top curve). (PNG) [file pone.0124303.s005.png]

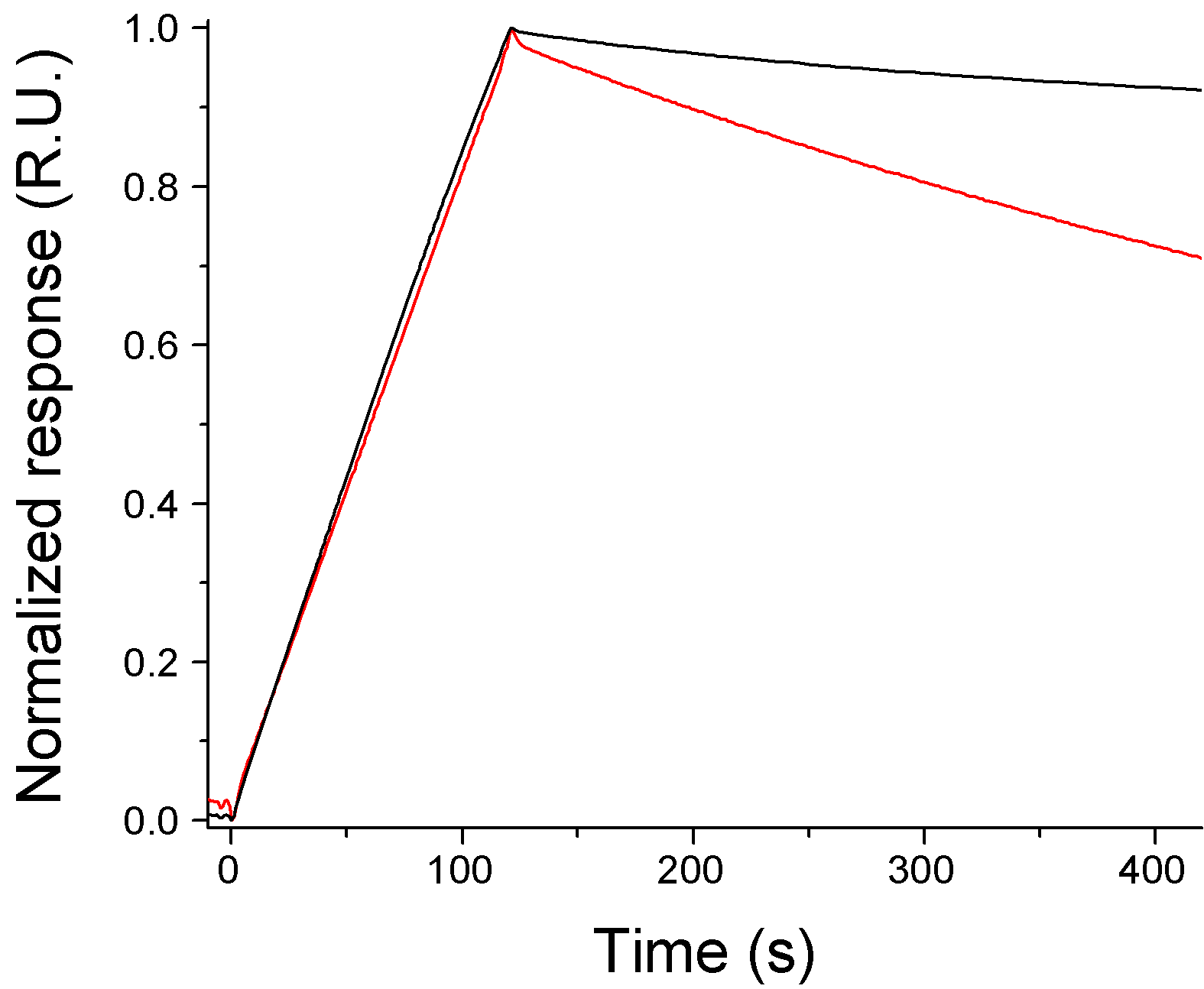

Supplement: S6 Fig — The normalized SPR sensorgrams show the interaction between a CM5 chip functionalized with anti-polyhistidine antibodies and the GFP-Nb (black line) and the his-eGFP (red line). Depicted sensorgrams were obtained for protein concentration of 35 nM. (PNG) [file pone.0124303.s006.png]

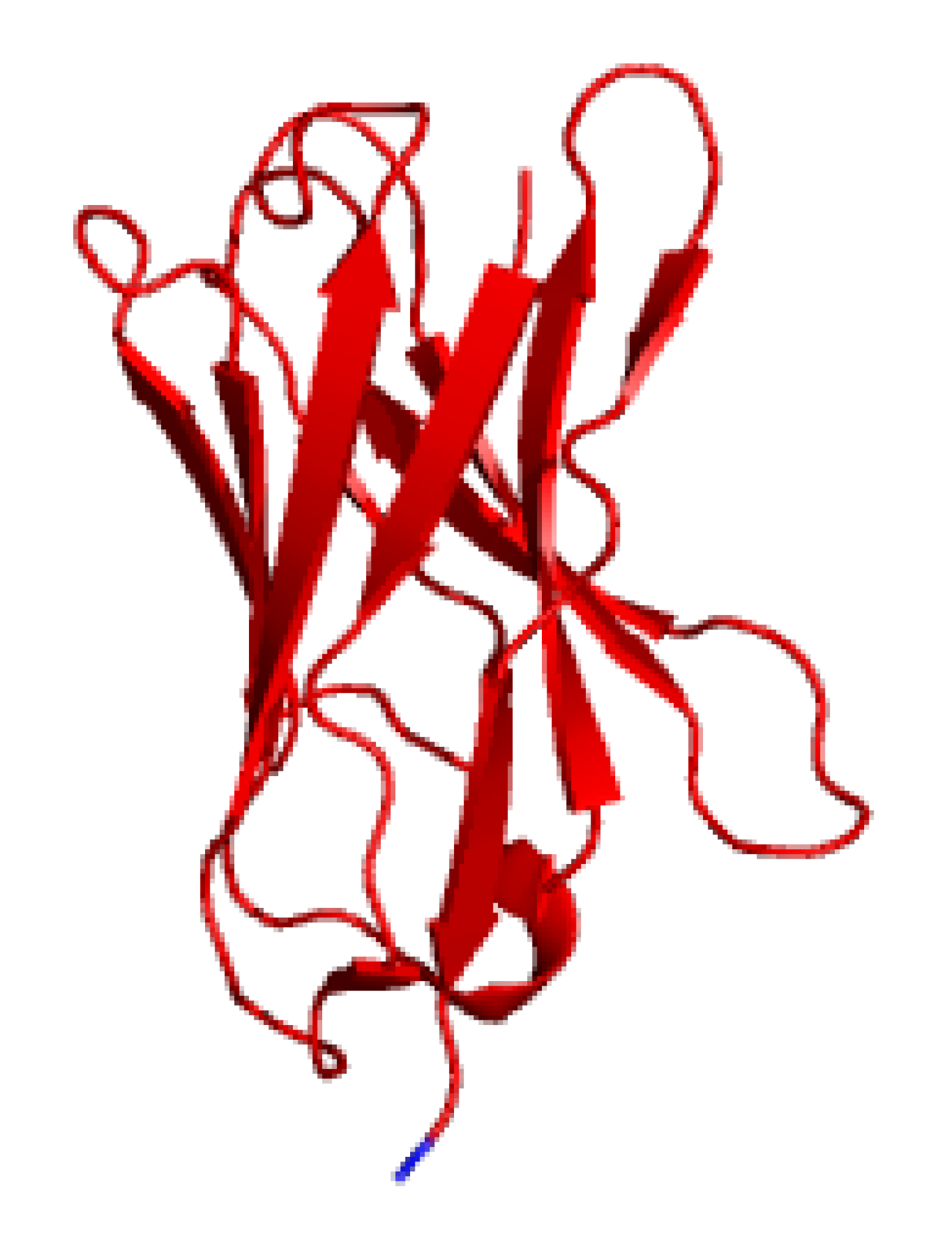

Supplement: S7 Fig — The protein ribbon structure is colored in red with the C-terminal-where the poly-histidine tag is inserted—shown in blue (PDB, 3OGO). (TIF) [file pone.0124303.s007.tif]

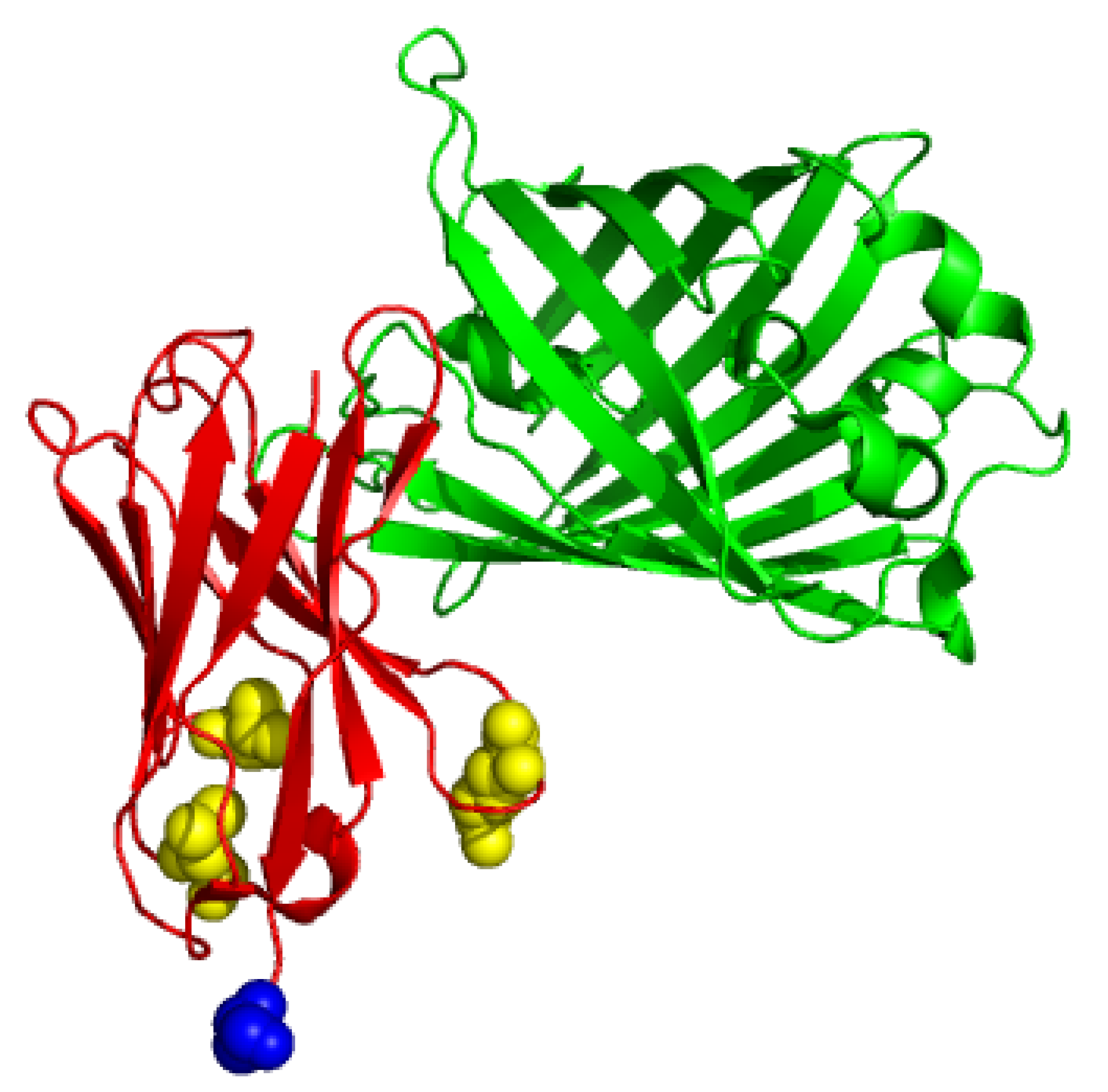

Supplement: S8 Fig — GFP ribbon structure is colored in green, the GFP-Nb is colored in red with the lysine residues highlighted as yellow spheres and the C-terminal highlighted as blue spheres (PDB, 3OGO). (TIF) [file pone.0124303.s008.tif]

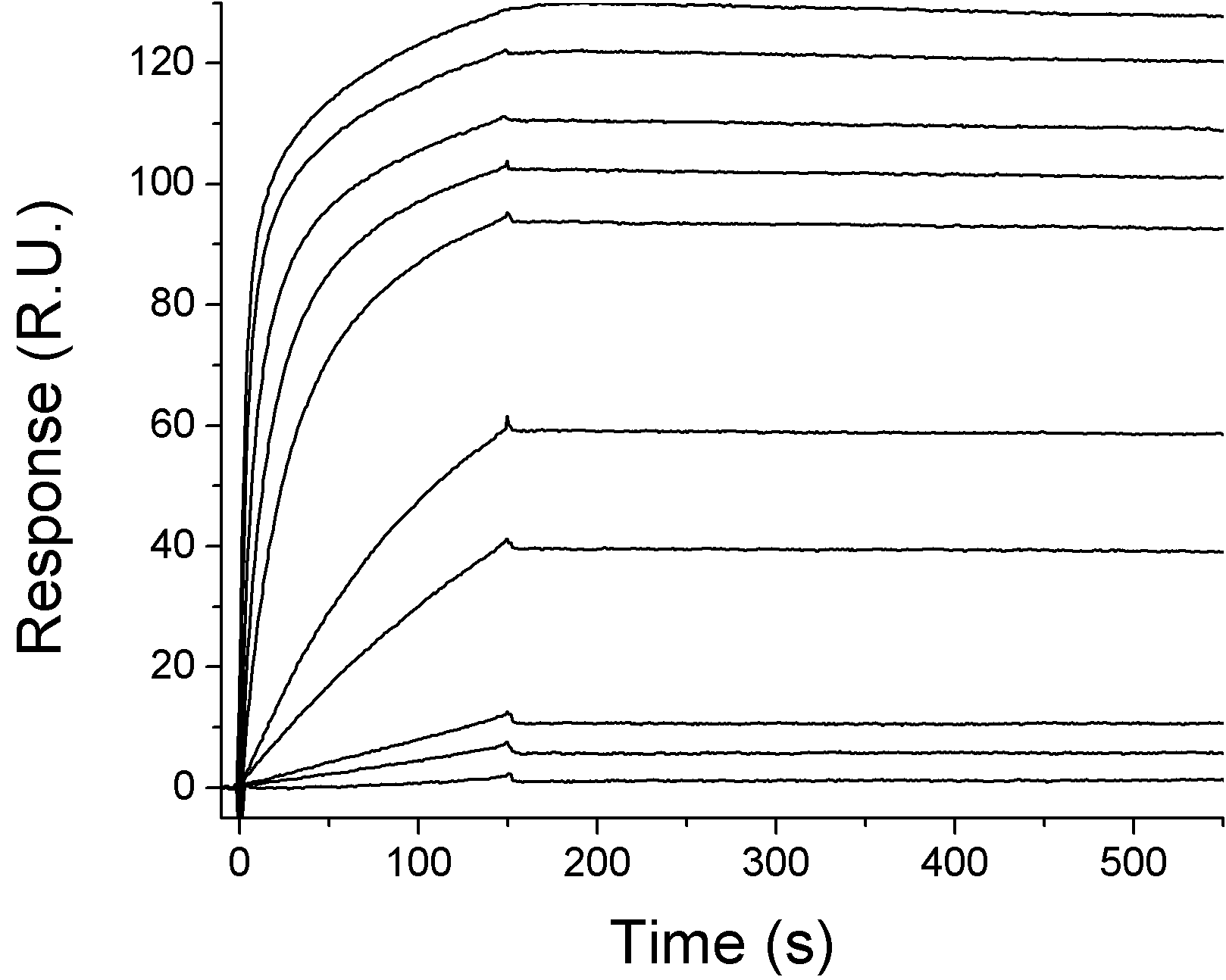

Supplement: S9 Fig — Binding of GFP to anti-GFP antibody immobilized on streptavidin on CAP chip. Depicted sensorgrams were obtained for GFP concentration of 0.1, 0.5, 1, 5, 10, 50, 100, 200, 500, 1000 nM (from bottom to top curve). (PNG) [file pone.0124303.s009.png]

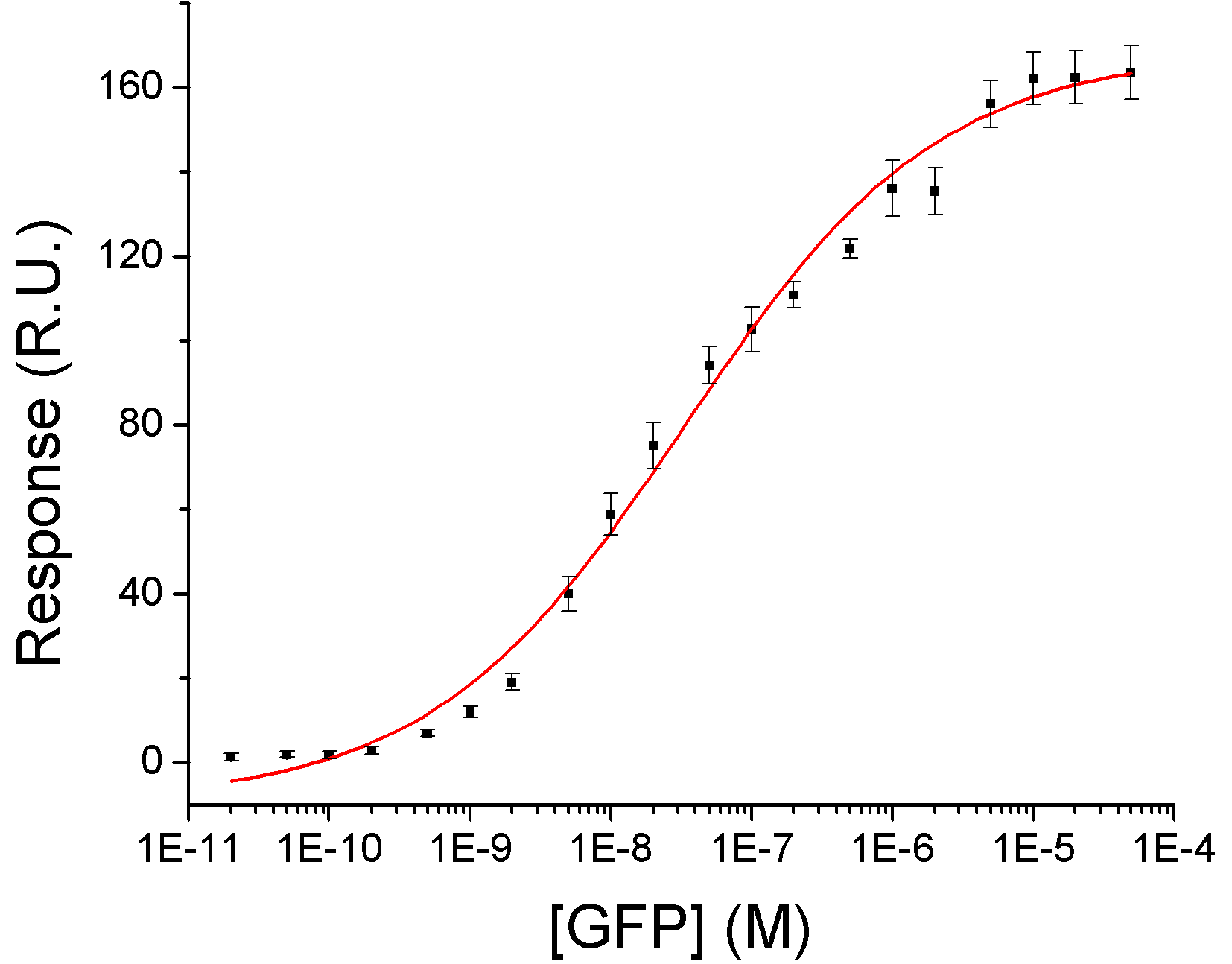

Supplement: S10 Fig — The squares are the raw data and the line is the best fit of the data set to the Hill’s equation (Hill’s coefficient set equal to 1). (PNG) [file pone.0124303.s010.png]

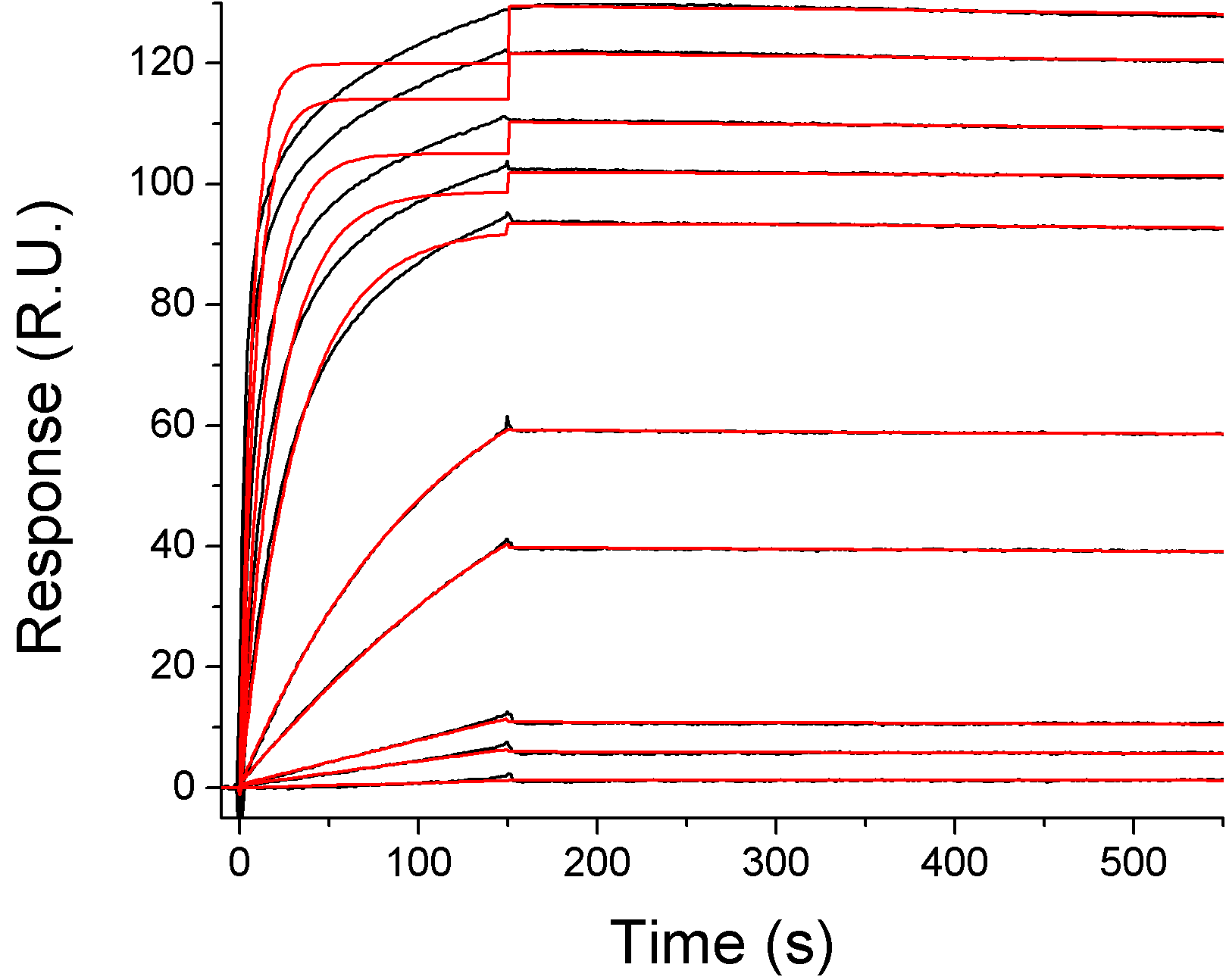

Supplement: S11 Fig — Binding of GFP to anti-GFP antibody immobilized on streptavidin on CAP chip. Black lines are raw data and the red lines are the fitting to a 1:1 binding model. Depicted sensorgrams were obtained for GFP concentration of 0.1, 0.5, 1, 5, 10, 50, 100, 200, 500, 1000 nM. (PNG) [file pone.0124303.s011.png]

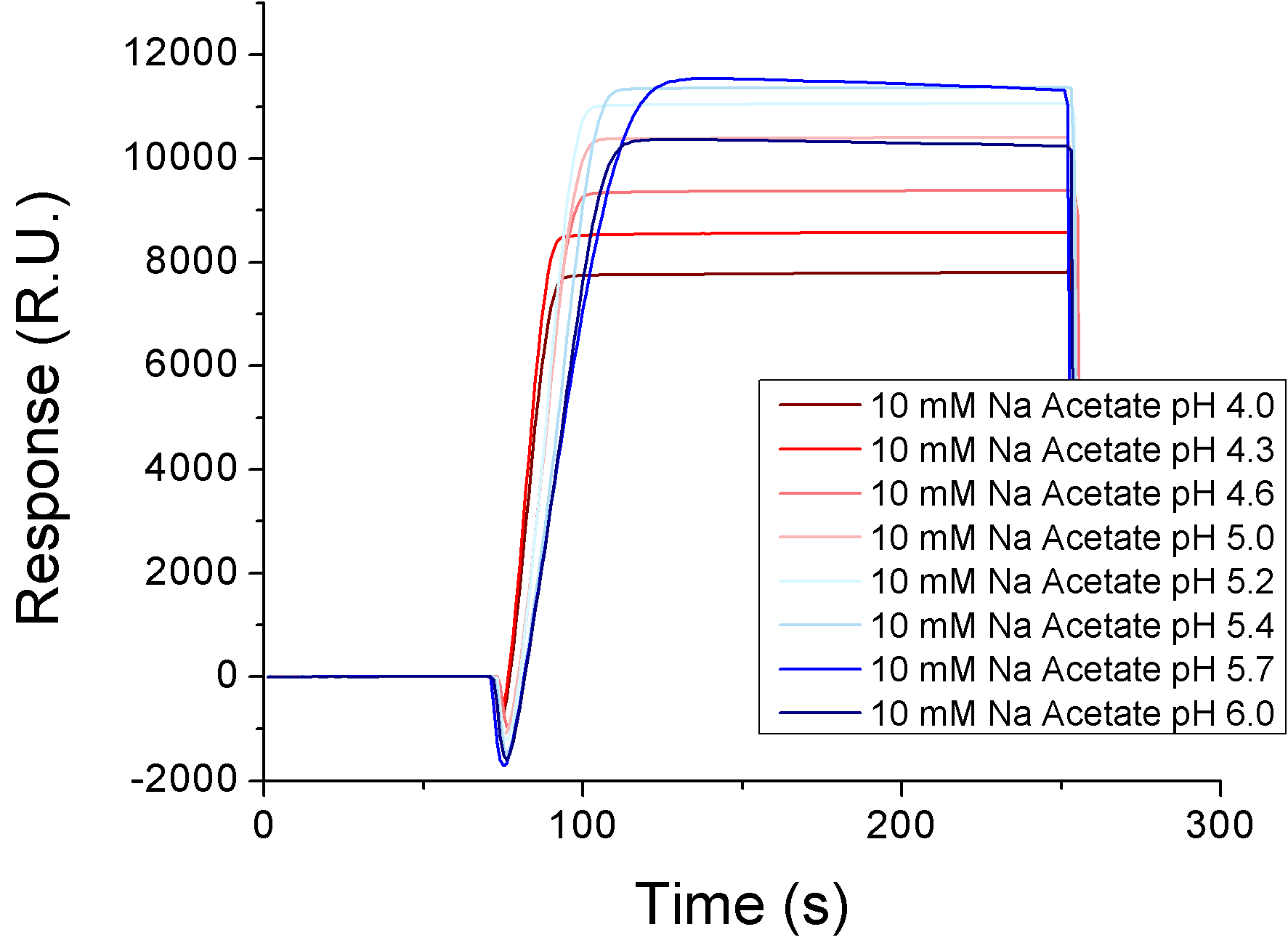

Supplement: S12 Fig — The protein was diluted in 10 mM sodium acetate buffer with pH varying between 4.0 and 6.0. The binding increases as the pH increases from 4.0 to 5.4. At pH higher than 5.7 the sensorgrams shows some dissociation after the initial binding. The bound nanobody dissociated completely from the surface at the end of the injection independently from the solution tested. The optimum pH for protein immobilization is 5.4. (PNG) [file pone.0124303.s012.png]

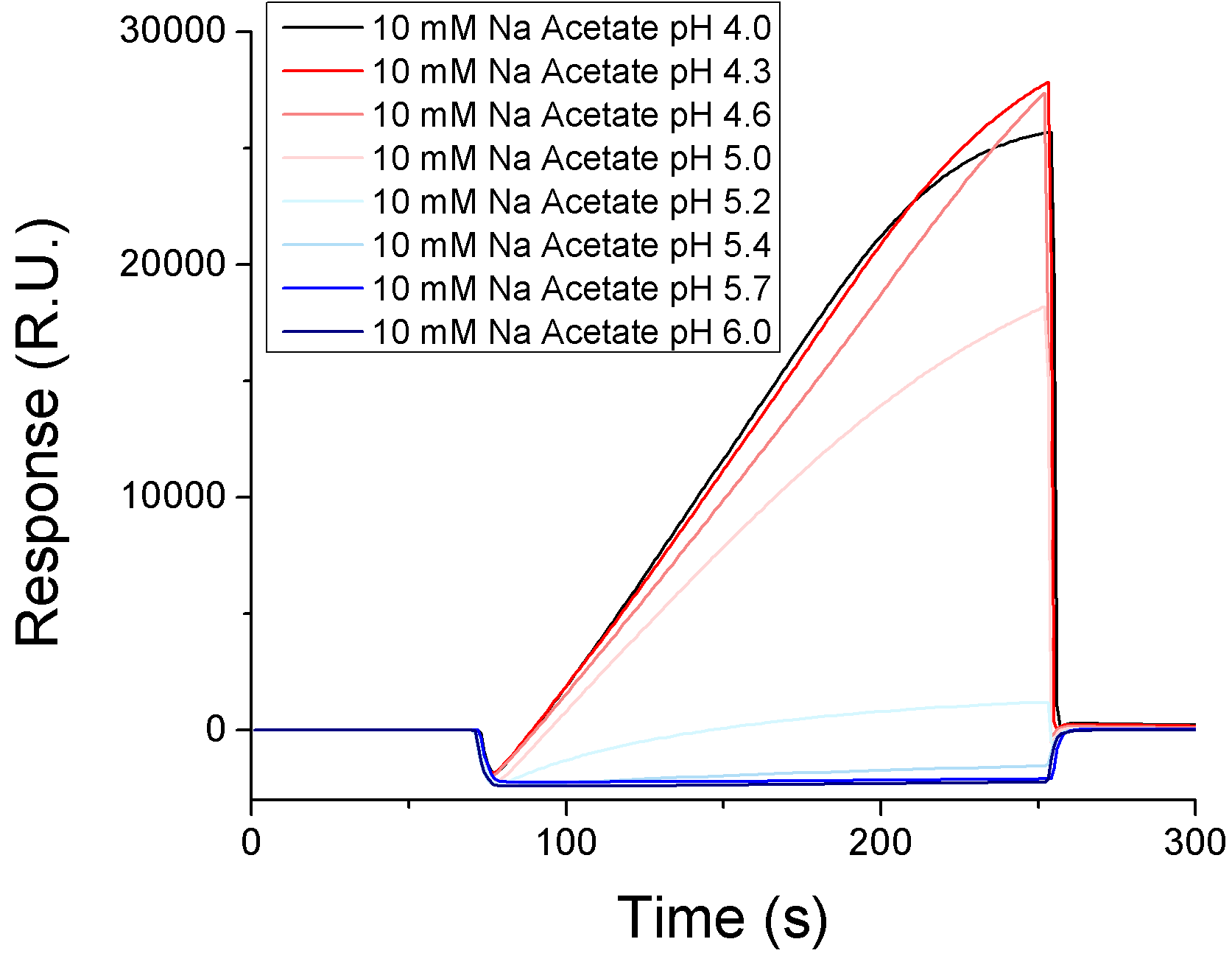

Supplement: S13 Fig — The protein was diluted in 10 mM sodium acetate buffer with pH varying between 4.0 and 6.0. The maximum binding is achieved at pH values between 4.3 and 4.6; the binding decreases for pH values higher than 4.6. The bound antibody dissociated completely from the surface at the end of the injection independently from the solution tested. The optimum pH for protein immobilization is 4.6. (PNG) [file pone.0124303.s013.png]

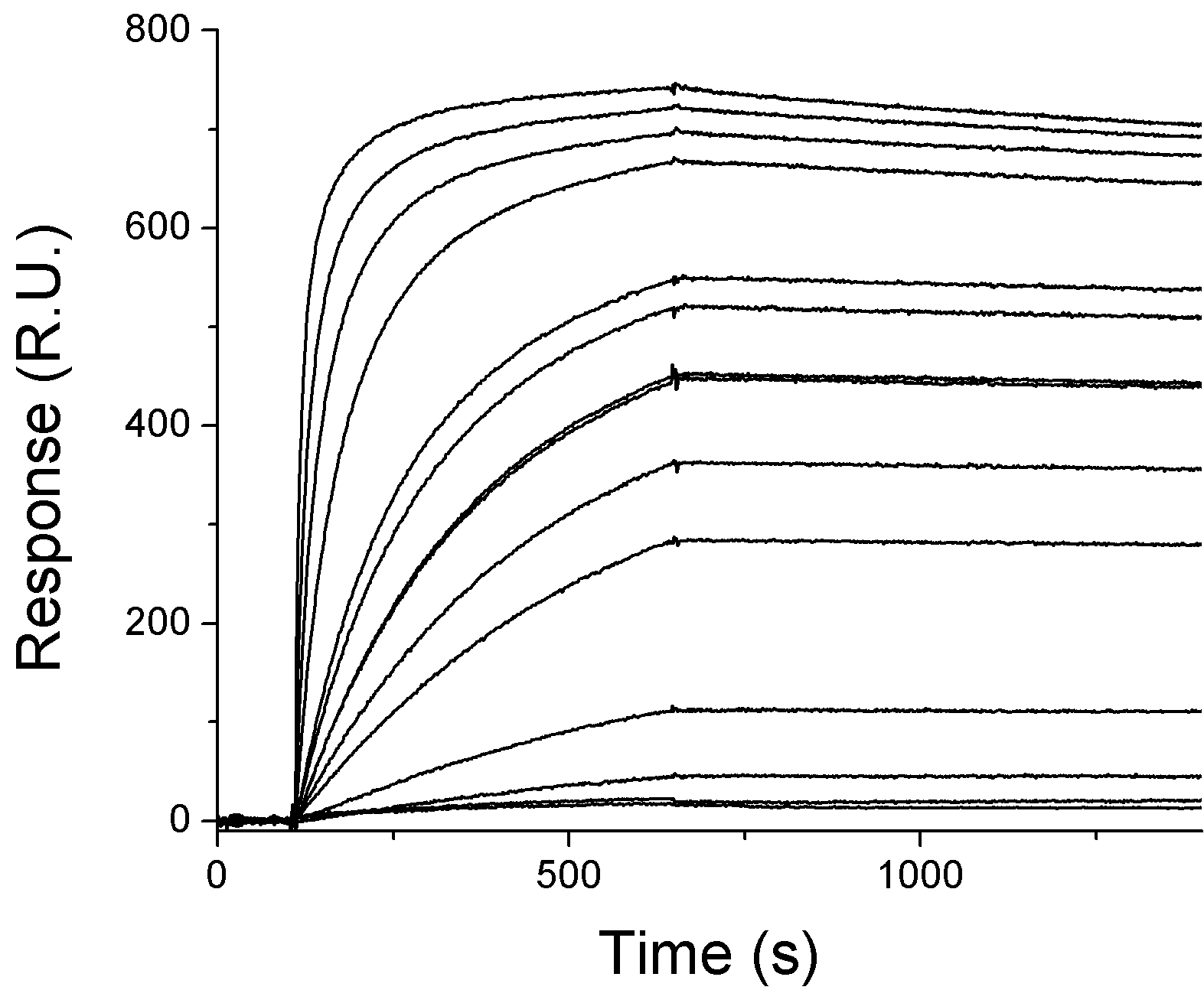

Supplement: S14 Fig — Depicted sensorgrams were obtained for GFP-Nb concentration of 0.2, 0.5, 1, 2, 5, 7, 10, 15, 20, 50, 100, 200, 500, 1000 nM (from bottom to top curve). (PNG) [file pone.0124303.s014.png]

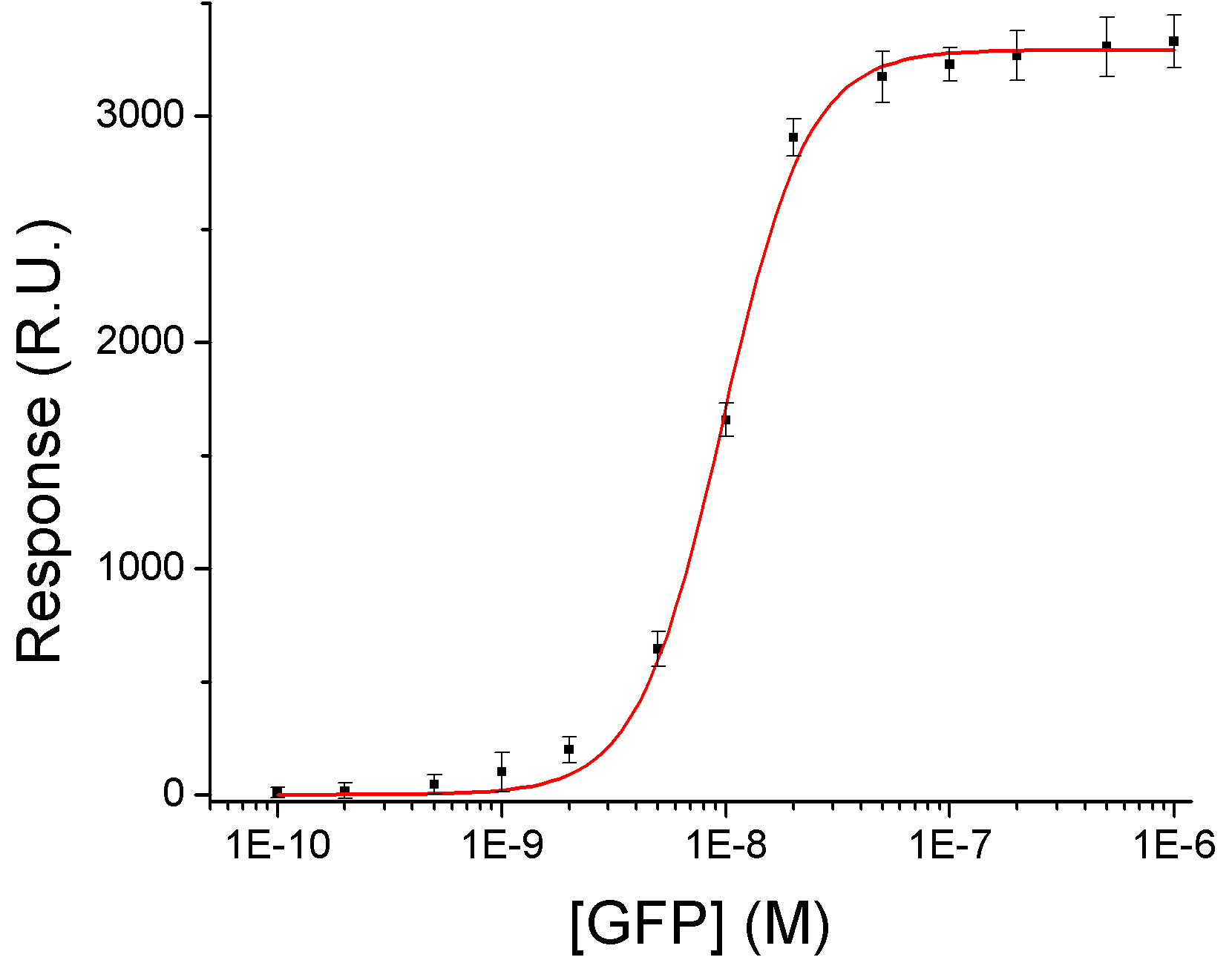

Supplement: S15 Fig — GFP-Nb was immobilized onto a CM5 chip via amino-coupling. The squares are the raw data and the line is the best fit of the data set to the Hill’s equation (Hill’s coefficient set equal to 1). (PNG) [file pone.0124303.s015.png]

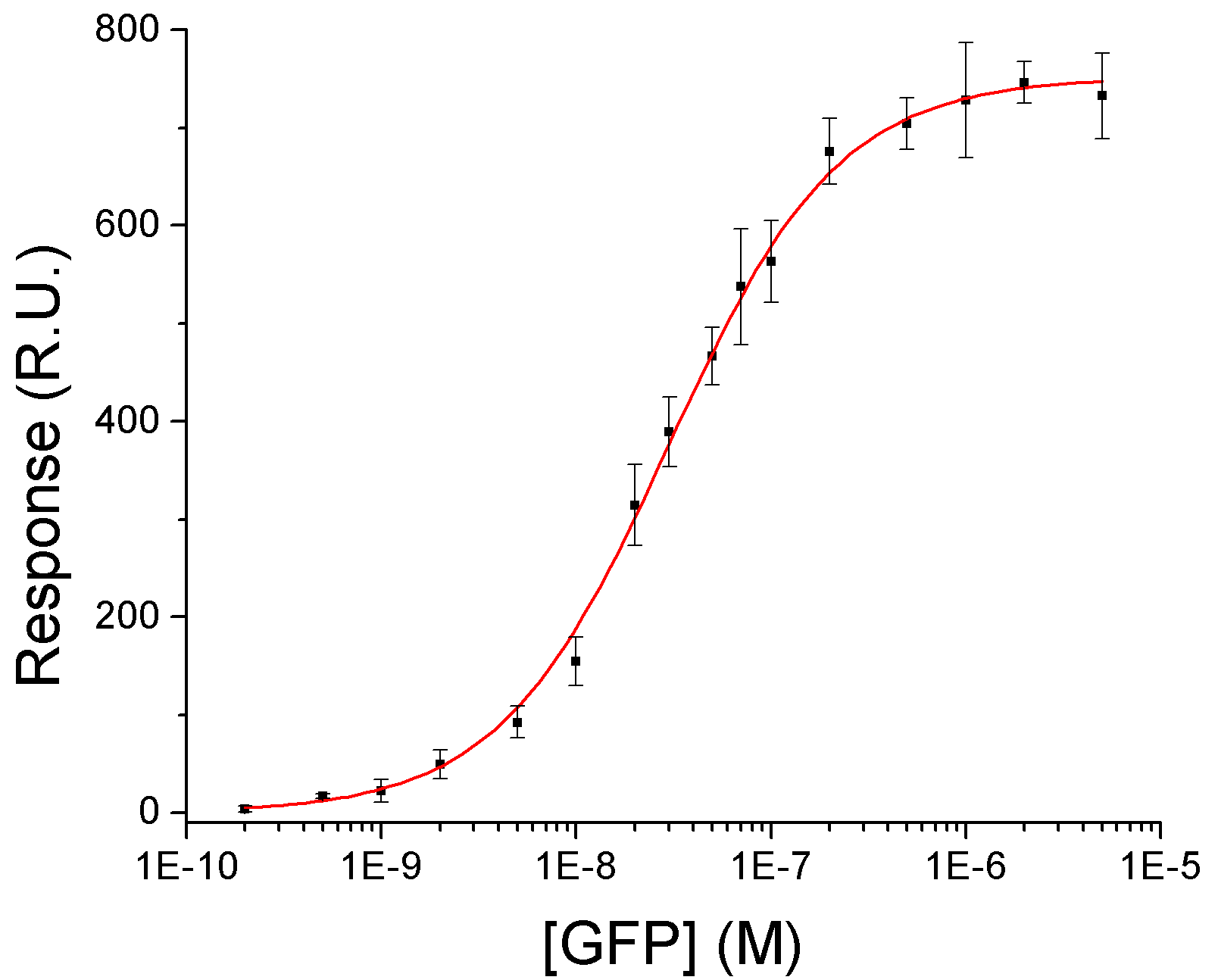

Supplement: S16 Fig — Anti-GFP was immobilized onto a CM5 chip via amino-coupling. The squares are the raw data and the line is the best fit of the data set to the Hill’s equation (Hill’s coefficient set equal to 1). (PNG) [file pone.0124303.s016.png]

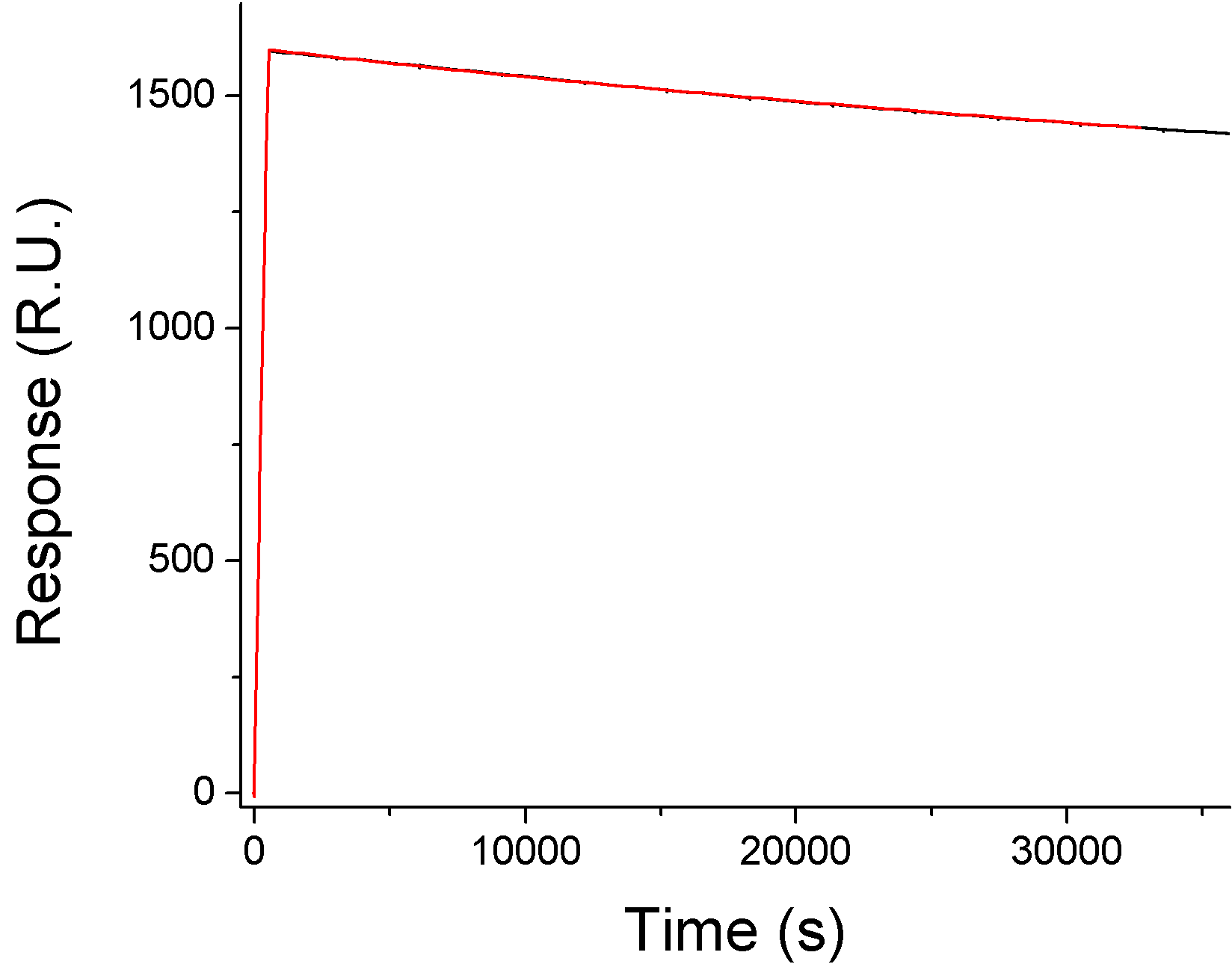

Supplement: S17 Fig — The sensorgrams show a run of immobilization of the GFP (10 nM) on the CM5 surface functionalized with the GFP-Nb. Dissociation of the GFP was followed for 36000 s. The black curve is the raw data and the red line is the fitting to a 1:1 binding model. (PNG) [file pone.0124303.s017.png]

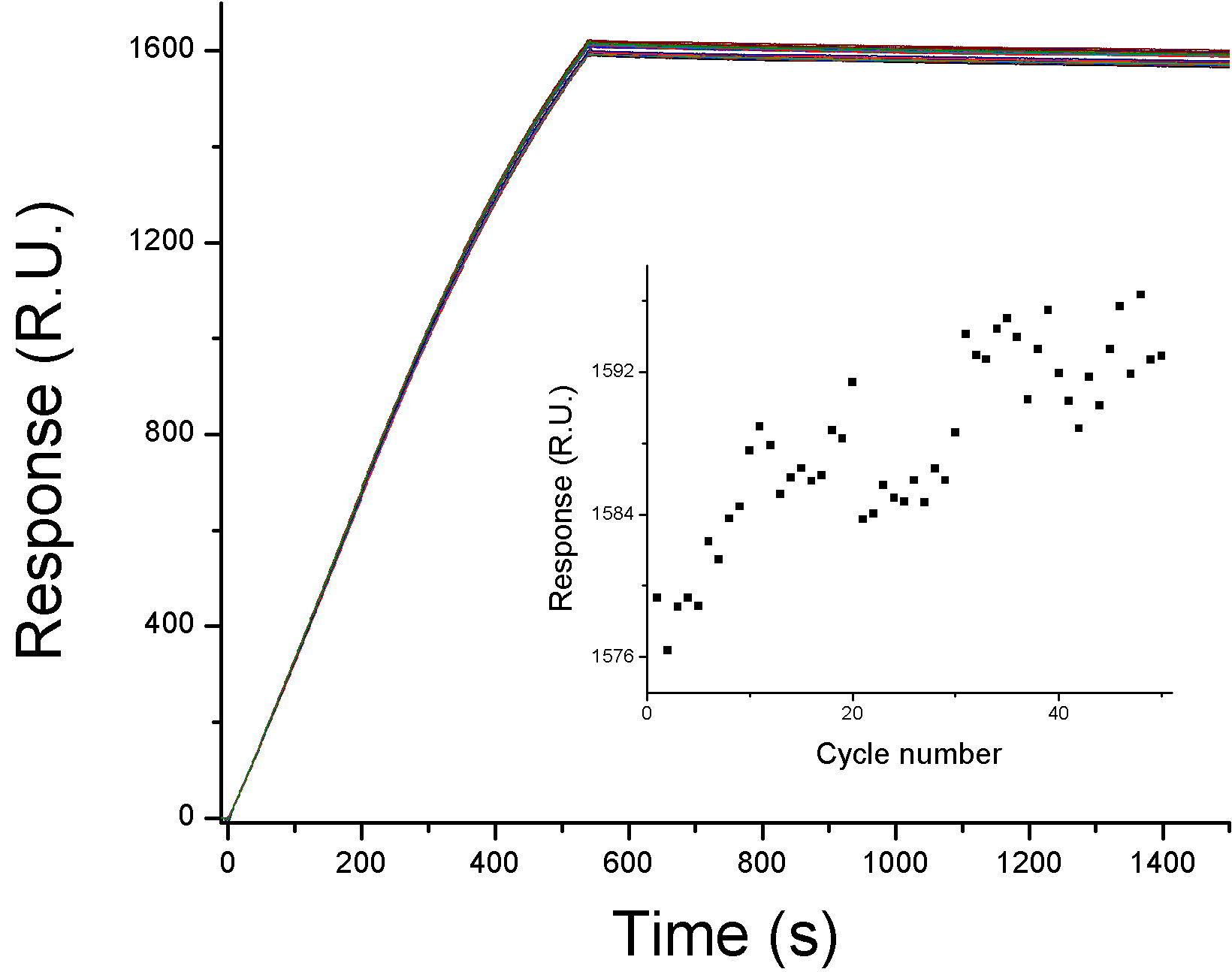

Supplement: S18 Fig — The sensorgrams show 50 runs of immobilization of the GFP (10 nM) on the CM5 surface functionalized with the GFP-Nb. The inset shows the Rmax value as a function of the cycle number. (PNG) [file pone.0124303.s018.png]

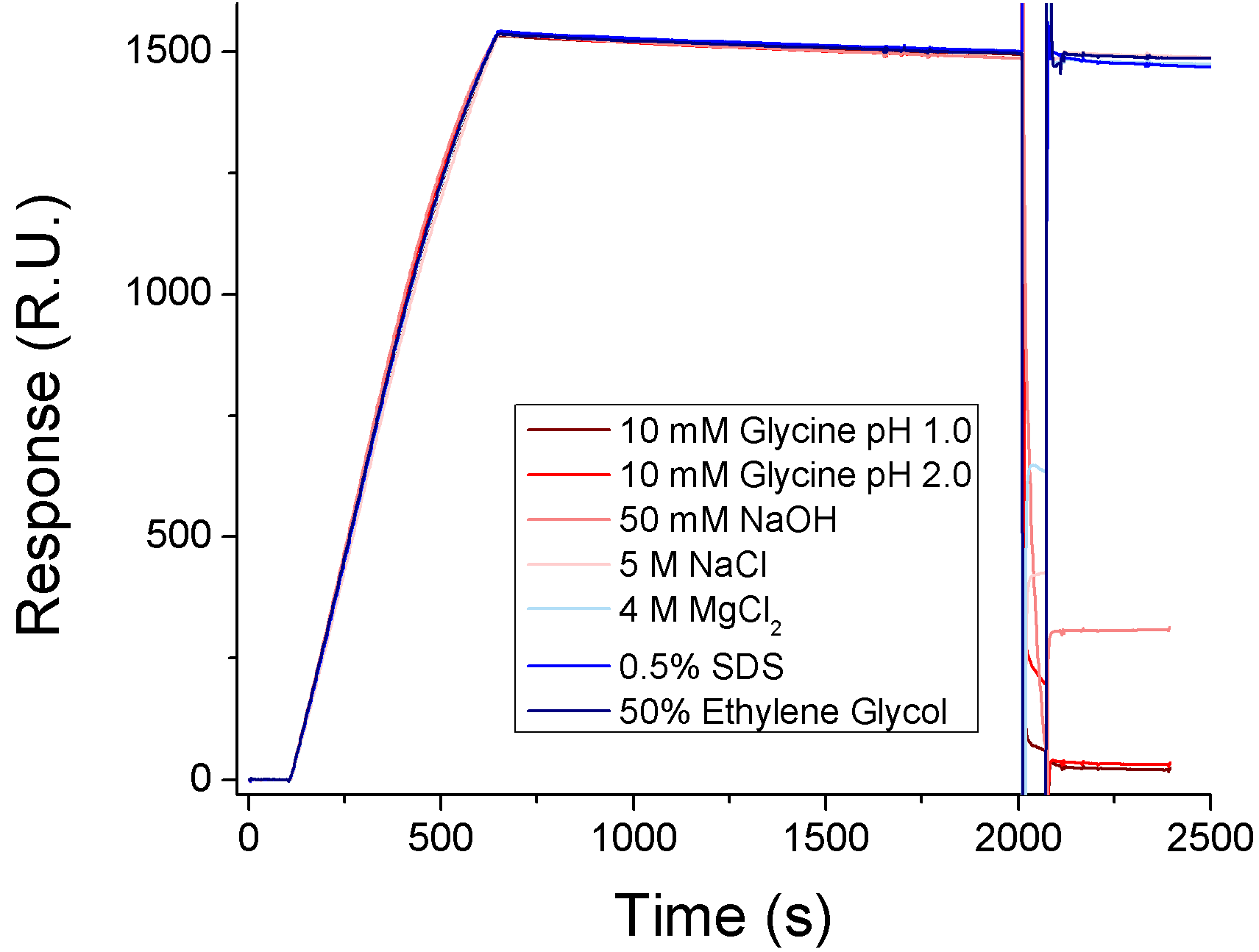

Supplement: S19 Fig — The sensorgrams show the immobilization of 10 nM GFP on a CM5 surface functionalized with the GFP-Nb followed by regeneration with a series of solutions. While 10 mM glycine solution removed completely the GFP from the surface and 50 mM NaOH removed partially the GFP, all the other solutions did not affect the binding of GFP to the immobilized nanobody. (PNG) [file pone.0124303.s019.png]

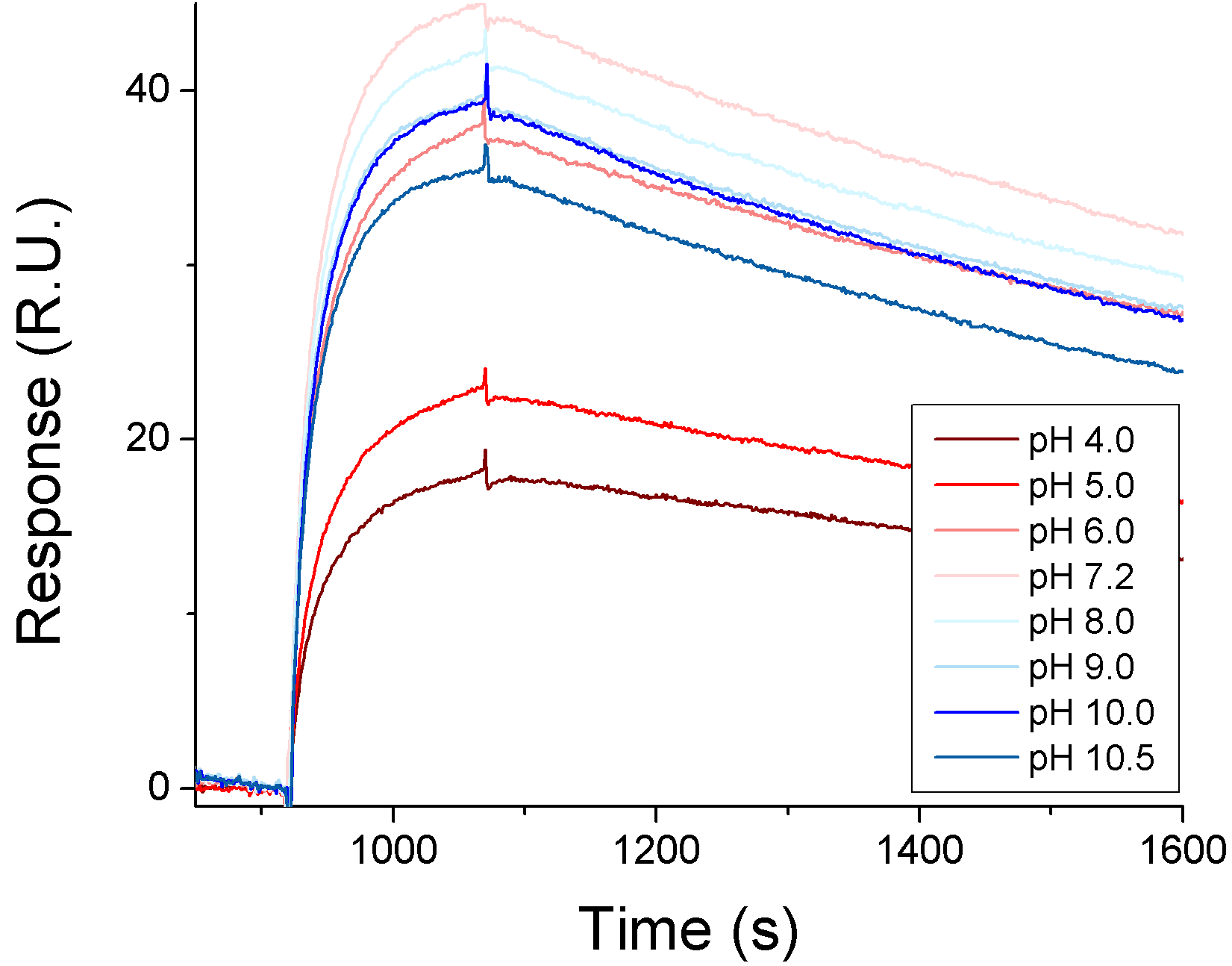

Supplement: S20 Fig — The sensorgrams illustrates the binding of 30 nM GFP to the GFP-Nb immobilized on a Ni:NTA surface. Before immobilization, the nanobody was diluted in NTA running buffer of several pH (range 4.0–10.5, see legend) and then applied to the Ni:NTA surface. (PNG) [file pone.0124303.s020.png]

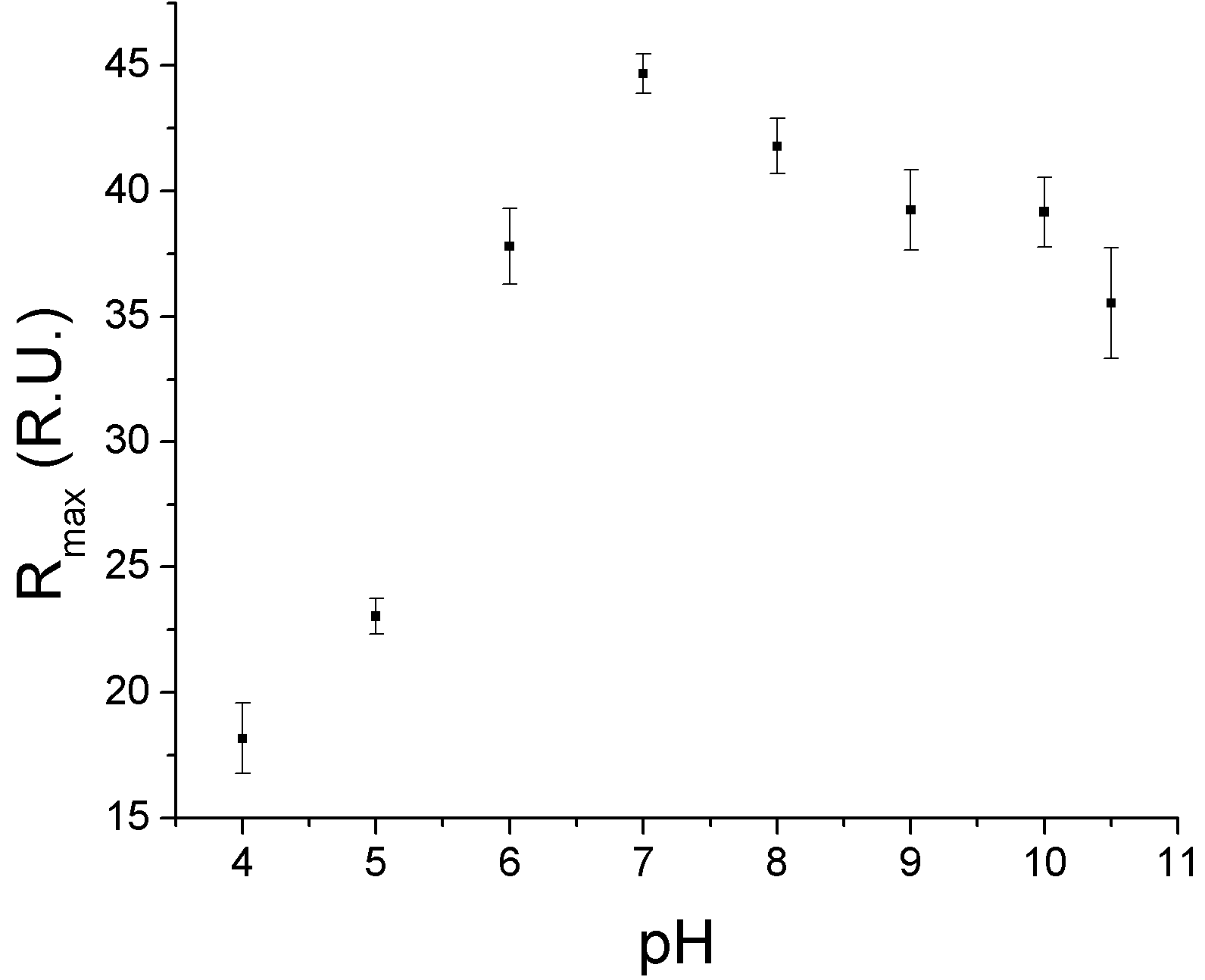

Supplement: S21 Fig — The plot shows the maximum response of the Ni:NTA chip for the nanobody as a function of the solution pH. (PNG) [file pone.0124303.s021.png]

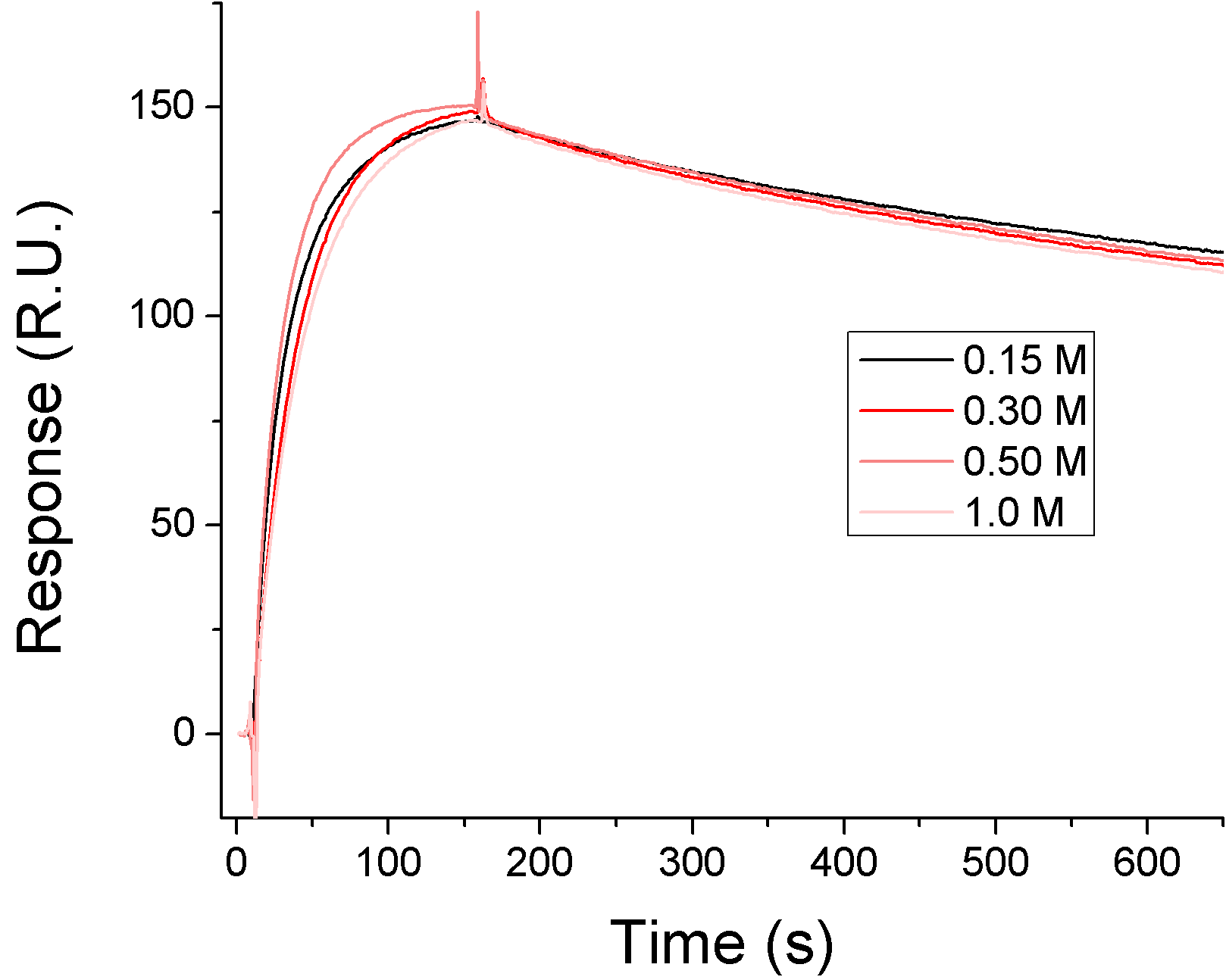

Supplement: S22 Fig — The sensorgrams illustrates the binding of the GFP-nanobody immobilized on a Ni:NTA surface to 30 nM GFP diluted in NTA running buffer with different concentration of NaCl (range 0.15 M-1.0 M, see legend). (PNG) [file pone.0124303.s022.png]
